# Developing and experimentally validating a glucocorticoid signaling-related gene signature to evaluate the prognosis and immunotherapeutic response in kidney renal clear cell carcinoma

**DOI:** 10.1371/journal.pone.0334104

**Published:** 2025-10-13

**Authors:** Yu Zhang, Chen Chen, Tianhang Zhu, Wei Luo, Ranran Zhou, Wanlong Tan

**Affiliations:** 1 Department of Urology, Nanfang Hospital, Southern Medical University, Guangzhou, Guangdong, China; 2 Department of Urology, Shenzhen Third People’s Hospital, Shenzhen, Guangdong, China; 3 Department of Urology, The Third Affiliated Hospital of Southern Medical University, Guangzhou, Guangdong, China; OMICS, PERU

## Abstract

Glucocorticoids play a pivotal role in tumorigenesis and cancer progression. However, the prognostic significance of glucocorticoid signaling-related genes remains poorly understood, particularly in kidney renal clear cell carcinoma (KIRC). Collected samples indicated KIRC patients exhibited elevated serum glucocorticoid levels compared to healthy donors (*P* < 0.05). Glucocorticoid signaling-related genes were curated from the MSigDB database. The TCGA-KIRC cohort was utilized for training, while 7 independent public KIRC cohorts and local samples were employed for validation. Through LASSO and random forest analyses, ACADM, ANGPTL4, and NFKB2 were identified and subsequently incorporated into a multivariate Cox regression model. This gene signature emerged as a robust prognostic indicator across multiple cohorts (pooled hazard ratio [HR] = 2.73, 95% confidence interval [CI] = 2.05–3.65). In local samples, KIRC tissues exhibited increased infiltration of NFKB2+ cells and decreased levels of ACADM+ and ANGPTL4+ cells (all *P* < 0.05). Meta-analyses and spatial transcriptomics revealed a positive association between the signature and CD8+ T cell infiltration. Furthermore, the signature was associated with T cell exhaustion levels and could predict immunotherapeutic responses in both computational simulations and real-world clinical settings (all *P* < 0.05). In vivo experiments showed that NFKB2 knockdown inhibited tumor growth and the expansion of CD8+PDCD1+ T cells, effects that were reversible with corticosterone treatment (all *P* < 0.05). Collectively, a glucocorticoid signaling-related gene signature was developed and rigorously validated as a predictive tool for prognosis and immunotherapeutic response in KIRC, offering valuable insights for guiding personalized treatment strategies.

## Introduction

Kidney renal clear cell carcinoma (KIRC) is the most common subtype of renal cell carcinoma, accounting for approximately 70–80% of cases, with a rising global incidence [1]. Despite significant advancements in therapeutic strategies, including targeted therapies such as tyrosine kinase inhibitors (TKIs) and immune checkpoint inhibitors (ICIs), the prognosis for patients with KIRC remains suboptimal, with a high rate of recurrence and metastasis [2]. The inherent heterogeneity of KIRC, coupled with the development of resistance to conventional therapies, underscores the urgent need for a deeper understanding of the molecular mechanisms driving disease progression. Recent studies have highlighted the critical roles of genetic mutations, epigenetic alterations, and dysregulated signaling pathways, such as the VHL/HIF-α axis, PI3K/AKT/mTOR, and Wnt/β-catenin, in promoting tumorigenesis, angiogenesis, and immune evasion in KIRC [3,4]. However, the complexity of these mechanisms and the lack of reliable biomarkers for prognosis prediction continue to pose significant challenges in clinical management [5,6]. Consequently, there is a pressing demand for the identification of novel biomarkers that can accurately predict patient outcomes and guide personalized therapeutic interventions. These biomarkers not only hold promise for improving prognostic stratification but also offer potential targets for the development of innovative, precision-based therapies, thereby addressing the unmet clinical needs in KIRC treatment.

Glucocorticoids (GCs), a class of steroid hormones, are crucial regulators of diverse physiological processes such as metabolism, immune response, and cell proliferation [7]. In the context of tumor biology, GCs exhibit a dual role, demonstrating both anti-tumor and pro-tumor effects depending on the specific conditions. They are widely utilized in cancer therapy to mitigate inflammation, alleviate chemotherapy-induced side effects, and induce apoptosis in certain hematologic malignancies, including lymphomas and leukemias [8]. Conversely, in various solid tumors, GCs may facilitate tumor growth, metastasis, and therapy resistance by suppressing immune responses and activating cell survival pathways [9,10]. These effects are mediated through the binding of GCs to glucocorticoid receptor (GR), which subsequently translocate to the nucleus to modulate gene expression, including genes associated with anti-inflammatory responses and cell survival [11]. Notably, it has been reported that GR promotes the proliferation of KIRC cells via the activation of STAT5, highlighting the pro-tumorigenic role of GCs in KIRC [12]. Despite these findings, the levels of GCs in KIRC patients remain unreported, and the prognostic significance of GC signaling-related genes in KIRC is largely unexplored. Elucidating the prognostic value of GC signaling-related genes not only helps guide personalized treatment but also facilitates the development of therapeutic drugs targeting this pathway in KIRC.

This study is methodically organized into three distinct phases to ensure a comprehensive investigation. In the initial phase, peripheral blood samples were collected from both KIRC patients and healthy donors at our local hospital. The serum concentrations of GCs were quantified using enzyme-linked immunosorbent assay (ELISA). In the second phase, genes associated with GCs signaling were curated from the Molecular Signatures Database (MSigDB). The KIRC cohort from The Cancer Genome Atlas (TCGA) served as the training dataset, while seven independent KIRC cohorts obtained from the Gene Expression Omnibus (GEO), ArrayExpress, and previously published studies were employed as validation datasets. Feature selection was performed using the least absolute shrinkage and selection operator (LASSO) regression and random forest analysis, followed by multivariate Cox regression to develop a robust risk signature. To validate our findings, paracarcinoma and KIRC tissue samples from our local hospital were analyzed. The association of the risk signature with CD8+ T cell infiltration and T cell exhaustion (TEX) levels was evaluated to account for the unfavorable prognosis of KIRC subjects with high risk. Additionally, the predictive ability of the risk signature for immunotherapeutic response in KIRC subjects was assessed across multiple cohorts. In the final phase, in vivo experiments were conducted using BALB/c mice to investigate the impact of the identified gene on tumor growth and TEX. Furthermore, the reverse effects of GCs were explored by treating the mice with corticosterone (CORT).

## Materials and methods

### Data collection and processing

To construct the training cohort, we obtained RNA sequencing (RNA-seq) data matrices comprising 72 paracarcinoma and 535 KIRC samples, along with their corresponding clinicopathological features and overall survival (OS) data, from TCGA database (https://portal.gdc.cancer.gov/). For external validation, we retrieved transcriptome sequencing data from five independent datasets (GSE29609 [13], GSE167573 [14], GSE53757 [15], GSE73731 [16], and GSE40435 [17]) through the GEO repository (https://ncbi.nlm.nih.gov/geo/). For the GEO-derived microarray datasets generated from diverse platforms, normalization was performed using R software. Background correction and quantile normalization were first conducted with the limma package, and the raw CEL files were uniformly processed using the RMA algorithm from the affy package. Additionally, intra-batch outlier samples (with PCA distances > 6 SD) were excluded, while high-quality data exhibiting inter-sample correlation coefficients > 0.85 were retained. Furthermore, transcriptome sequencing data from the E-MTAB-1980 cohort [18] were acquired from the ArrayExpress database (https://www.ebi.ac.uk/biostudies/arrayexpress). To specifically evaluate treatment response, we incorporated the JAVELIN Renal 101 cohort, which includes RNA-seq data from 726 KIRC samples with corresponding progression-free interval (PFI) and treatment response data for avelumab plus axitinib combination therapy, obtained from supplementary materials of the study by Motzer et al [19]. The baseline clinicopathological characteristics of the KIRC cohorts are summarized in S1 Table. Except for the TCGA-KIRC and GSE73731 cohorts, for which preoperative medication history was not available, none of the other cohorts had received any form of treatment, including radiotherapy, chemotherapy, or immunotherapy, prior to surgery. Additionally, literature review confirmed that these cohorts were independent. The RNA-seq matrices and OS duration of the subjects with other 32 cancer types were also downloaded from TCGA for pan-cancer analyses. All public datasets underwent rigorous normalization and quality control procedures, with detailed methodology documented in S1 File. Additionally, a sum of 655 GC signaling-related genes was collected from the MSigDB (https://www.gsea-msigdb.org/gsea/msigdb/index.jsp), with annotation details provided in S2 Table.

### Unsupervised clustering

Non-negative matrix factorization (NMF) clustering was performed using the NMF R package, with the optimal cluster number determined by cophenetic correlation, dispersion coefficients, and silhouette scores.

### Differential gene expression analysis

Differential gene expression analysis between the two groups was conducted using the edgeR package, identifying statistically significant differentially expressed genes (DEGs) with an absolute log2 fold change (|log2FC|) > 1 and a false discovery rate (FDR) adjusted *P*-value < 0.05 [20]. The count format of the matrix was adopted.

### Protein-protein interaction (PPI) network construction and functional enrichment

A PPI network was constructed using the STRING database (https://string-db.org/) with a medium confidence interaction score threshold of 0.4. Functional enrichment analysis was performed using the Metascape platform (https://metascape.org/), with enrichment terms considered significant at a nominal *P*-value < 0.05 and a Benjamini-Hochberg adjusted *P*-value (q-value) < 0.05.

### Single cell analysis and public immunohistochemistry (IHC) staining analysis

Single-cell level analyses were conducted using the CancerSEA database (http://biocc.hrbmu.edu.cn/CancerSEA/) to explore the correlation between gene expression profiles and critical malignant phenotypes in KIRC samples. The IHC staining of the identified genes in normal kidney and KIRC tissues was obtained from The Human Protein Atlas (HPA, https://www.proteinatlas.org/). Quantitative analysis of the IHC-stained tissue sections was performed using Image-Pro Plus 6.0 software.

### Single-sample gene set enrichment analysis (ssGSEA) and deconvolution algorithms

The activation status of GC signaling and immune-related pathways was assessed using ssGSEA with the GSVA package, and immune cell infiltration levels were quantified using the CIBERSORT-ABS algorithm, further validated by five additional deconvolution algorithms (TIMER, CIBERSORT, QUANTISEQ, MCP-COUNTER, and xCELL) to evaluate CD8+ T cell infiltration.

### Assessment of immunotherapy and chemotherapy efficacy

The therapeutic response of KIRC patients to ICIs was assessed using the tumor immune dysfunction and exclusion (TIDE) algorithm (http://tide.dfci.harvard.edu/). Finally, drug sensitivity prediction was conducted using the oncoPredict R package, evaluating the half-maximal inhibitory concentration (IC50) values of KIRC subjects in response to various chemotherapeutic agents based on the Genomics of Drug Sensitivity in Cancer (GDSC) database.

### Risk model construction and meta-analyses

To identify genes significantly associated with OS in the TCGA-KIRC cohort, we employed a dual-feature selection approach using LASSO regression and random forest algorithms, implemented via the glmnet and RandomForestSRC R packages, respectively. LASSO efficiently selects the most relevant linear features and reduces dimensionality, while Random Forest captures complex, non-linear interactions and ranks feature importance, so combining them leverages both linear and non-linear advantages for more robust feature selection [21]. Genes consistently identified by both methods were used to construct a multivariate Cox regression model, from which a GC signaling-related score (GSRS) was calculated for each patient. GSRS was calculated as follows: $GSRS=\sum_{i=\text{1}}^{n}({Coefficient}_{i}*{exp(gene)}_{i}$, where “Coefficient” represents the regression coefficient of each gene in the multivariate Cox regression model, and “exp(gene)” denotes the corresponding gene mRNA expression level.

Additionally, meta-analyses were conducted using the meta R package to synthesize effect estimates (hazard ratios [HRs], odds ratios [ORs], and correlation coefficients), with heterogeneity assessed via the Cochrane Q test, employing random-effects or fixed-effect models based on the presence or absence of significant heterogeneity.

### Spatial transcriptomics analysis

Spatial transcriptomics analysis was performed using the CROST platform (https://ngdc.cncb.ac.cn/crost/home) to investigate the spatial correlation between gene expression patterns and CD8+ T cell infiltration, utilizing data from the published dataset by Meylan M et al [22]. Cell type annotations and expressions of ACADM, ANGPTL4, NFKB2, and CD8A in KIRC samples were directly obtained from the CROST platform. Cell type annotations for the spatial transcriptomics data were obtained from matched scRNA-seq data [23,24]. To analyze the spatial correlation between GSRS and TEX, we additionally downloaded spatial transcriptomic data from KIRC samples published by Meylan M et al. via the same platform. Data preprocessing was conducted using the Seurat package in R, including filtering out low-quality spots (fewer than 200 genes detected or mitochondrial gene content >20%). The gene expression matrix was normalized using SCTransform to mitigate technical variation [25]. GSRS scores were computed for each spot using the established formula, while TEX status was evaluated based on combined CD8A and PDCD1 expression levels. All results were visualized with ggplot2.

### Validation using clinical samples

The study collected paracarcinoma tissues, KIRC samples, and peripheral blood samples from KIRC patients at Shenzhen Third People’s Hospital between 02/03/2024 and 08/11/2024. To ensure robust comparisons and minimize selection bias, we established a comparison cohort at a 1:2 ratio (KIRC patients to healthy donors) for evaluating serum GC level differences. Healthy donor samples were obtained from the Physical Examination Center of the same institution. Detailed inclusion and exclusion criteria for both KIRC patients and healthy donors are provided in S1 File. Considering the influence of diurnal fluctuations on serum GC levels, blood draws were performed in the morning (8:30–9:30) for all participants.

Serum GC levels were quantified using species-specific ELISA kits (for both human and mouse samples) following the manufacturers’ standardized protocols. Immunofluorescence analyses were conducted to assess the expression levels of the identified genes in both paracarcinoma and KIRC tissues. The protocol of this study has been reviewed and approved by the Ethics Committee of Shenzhen Third People’s Hospital (No. 2023-118-06; 2023/12/2). All participants have provided written informed consent to participate in this study and for their data to be published.

### *In vivo* experimental validation

Renca cells were obtained from the American Type Culture Collection (ATCC) and cultured in Dulbecco’s Modified Eagle Medium (DMEM) under standard conditions. To establish Nfkb2 knockdown models, Renca cells were transduced with lentiviral vectors encoding shRNA specifically targeting Nfkb2 (provided in S1 File). The knockdown efficiency was validated through real-time quantitative PCR (RT-qPCR), with the corresponding primer sequences detailed in S3 Table.

For *in vivo* experiments, twelve 6–7-week-old male BALB/c mice were randomly allocated into three experimental groups (n = 4 per group): Vector control (KD), KD-NFKB2, and CORT treatment. The Vector (KD) group received subcutaneous injections of Renca cells transfected with empty control plasmids, while the KD-NFKB2 group was injected with Nfkb2-knockdown Renca cells. The CORT treatment group received both Nfkb2-knockdown Renca cell injections and CORT supplementation (50 μg/mL) in their drinking water [26].

Animals were humanely euthanized upon meeting predefined endpoints: a tumor diameter ≥ 15 mm or severe distress/moribund signs (e.g., > 20% weight loss, lethargy, hunched posture, impaired mobility, ulceration). Euthanasia was performed immediately upon identification of endpoint criteria during daily health and behavior monitoring. No animals died prior to meeting these humane endpoints in this study. The experiment commenced tumor measurements on day 7 post-injection and concluded on day 31 when the first mouse reached the tumor size endpoint, necessitating euthanasia for all animals as planned. All twelve animals were euthanized at this terminal endpoint: one for reaching tumor size and the remaining eleven concurrently. The cause of death for all animals was humane euthanasia following the planned protocol.

Animal health (including body condition, tumor appearance, activity level) and behavior were assessed daily. Tumor volume was measured every three days starting on day 7. To minimize suffering and distress, animals were housed in standard, pathogen-free conditions with environmental enrichment. Analgesics were not administered due to potential interference with the tumor biology and stress pathways under investigation. Euthanasia was induced under deep isoflurane anesthesia followed by cervical dislocation to ensure death without recovery. Personnel received specific IACUC-approved training in animal care, handling, pain/distress recognition, and aseptic techniques. The study, approved by the Southern Medical University Animal Care and Use Committee (SMU-L2023318; 2023/12/27), adhered to the 3Rs principles (Replacement, Reduction, Refinement).

Serum CORT levels were quantified using ELISA. Immunofluorescence staining to assess CD8+PDCD1+ T cell infiltration and immunohistochemical staining to determine Nfkb2 expression levels in the subcutaneous tumors were also conducted.

### Statistical analyses

All statistical analyses were conducted using R software (version 4.3.0). For comparisons of continuous variables between groups, the Welch’s corrected t-test or Wilcoxon signed-rank test was employed. Categorical variables were compared using the Chi-square test or Fisher’s exact test. Differences in OS over the follow-up period were evaluated using Kaplan-Meier curves, with statistical significance assessed via the log-rank test or two-stage hazard rate comparison. Spearman correlation coefficients were calculated using the cor.test function in R. A two-sided *P*-value < 0.05 was considered statistically significant for all analyses, unless otherwise specified. Additional methodological details are provided in S1 File.

## Results

### KIRC subjects exhibited increased serum GC levels

**Fig 1:**
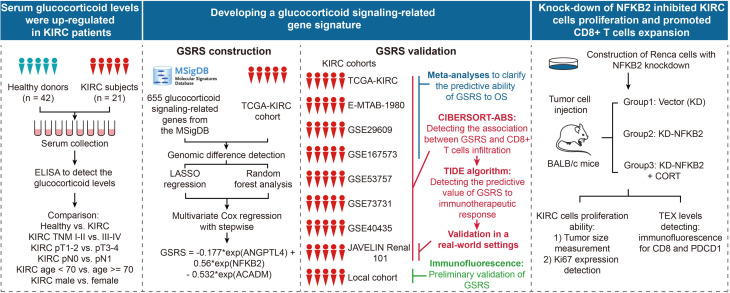
Schematic overview of the study workflow. The research framework comprised three principal components. First, serum glucocorticoid (GC) levels were quantified in both healthy donors and kidney renal clear cell carcinoma (KIRC) patients using enzyme-linked immunosorbent assay (ELISA), followed by comprehensive analysis of their correlation with critical clinicopathological parameters. Second, we established and rigorously validated a glucocorticoid signaling-related gene signature. This involved curating 655 glucocorticoid signaling-related genes from the Molecular Signatures Database (MSigDB, https://www.gsea-msigdb.org/gsea/msigdb/index.jsp) and utilizing the TCGA-KIRC cohort as the training dataset. Through the application of least absolute shrinkage and selection operator (LASSO) and random forest algorithms, we identified significant genes associated with overall survival (OS). A glucocorticoid signaling-related score (GSRS) was subsequently computed for each TCGA-KIRC patient using a multivariate Cox regression model. The predictive efficacy of GSRS for OS and immunotherapy response was then validated across seven independent KIRC cohorts and local patient samples. Finally, *in vivo* experiments were conducted using BALB/c mice implanted with Renaca cells, with or without NFKB2 knockdown, combined with corticosterone (CORT) treatment, to assess tumor cell proliferation and T cell exhaustion levels, thereby substantiating our findings.

**Fig 2:**
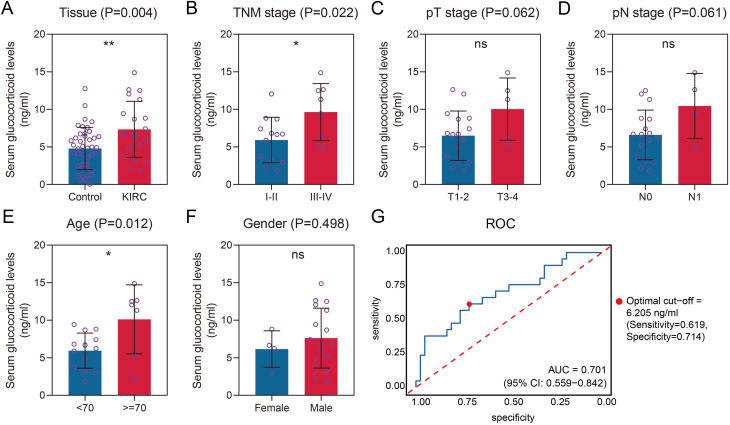
Elevated serum glucocorticoid levels in KIRC patients and their positive correlation with disease malignancy. A. Comparison of serum glucocorticoid levels between healthy donors and KIRC patients. B-F. Association of serum glucocorticoid levels with TNM stages (B), pathological T stages (C), pathological N stages (D), age (E), and gender (F). The optimal age stratification cut-off value was determined using X-tile software based on the TCGA-KIRC cohort. G. Receiver operating characteristic (ROC) analysis was performed to evaluate the diagnostic performance of serum GC in distinguishing between healthy donors and patients with KIRC. The optimal cut-off value was also identified.

The workflow of this study is schematically presented in Fig 1. In the initial phase, we employed ELISA to quantify serum cortisol levels, a predominant GC in humans, from peripheral blood samples obtained from 21 KIRC patients and 42 healthy donors recruited from our local hospital. The clinicopathological characteristics of the KIRC cohort are detailed in S1 Table. Comparative analysis revealed significantly elevated serum GC levels in KIRC patients compared to healthy controls (*P* < 0.01, Fig 2A). Furthermore, advanced-stage (TNM stage III-IV) KIRC patients demonstrated higher serum GC concentrations than early-stage (TNM stage I-II) cases (*P* < 0.05, Fig 2B). Subsequent association analyses were performed to examine the relationship between serum GC levels and various clinicopathological parameters of KIRC subjects. While no significant associations were observed with pathological T (pT) stage (*P* > 0.05, Fig 2C) or pathological N (pN) stage (*P* > 0.05, Fig 2D), a statistically significant positive association was identified with age (*P* < 0.05, Fig 2E). No significant gender-based differences were detected (*P* > 0.05, Fig 2F). The optimal age stratification threshold was determined using X-tile software analysis [27] of the TCGA-KIRC cohort. Receiver operating characteristic (ROC) analysis demonstrated that serum GC exhibited favorable diagnostic performance in distinguishing healthy donors from patients with KIRC, achieving an area under the curve (AUC) of 0.701 (95% confidence interval [CI] = 0.559–0.842). The optimal cutoff value, as determined by the ROC analysis, was 6.205 ng/mL, which corresponded to a sensitivity of 0.619 and a specificity of 0.714 (Fig 2G). Collectively, our findings demonstrate a significant upregulation of serum GC levels in KIRC patients, with a positive correlation observed between GC concentrations and the malignant progression of KIRC. These results strongly suggest that GCs play a pivotal role in both the tumorigenesis and disease progression of KIRC.

### 112 GC signaling-related genes were differentially expressed

**Fig 3:**
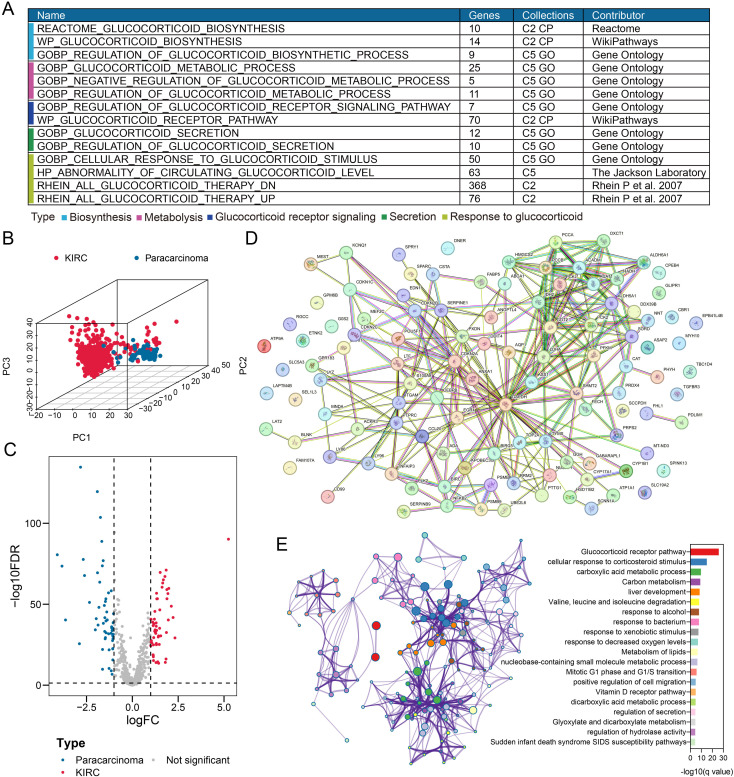
Differential expression of glucocorticoid signaling-related genes between paracarcinoma and KIRC tissues in the TCGA-KIRC cohort. A. A total of 655 glucocorticoid signaling-related genes were curated from the MSigDB database. B. Principal component analysis (PCA) revealed distinct expression patterns of glucocorticoid signaling-related genes in paracarcinoma and KIRC tissues. C. A volcano plot identified 112 out of 655 glucocorticoid signaling-related genes as differentially expressed between paracarcinoma and KIRC tissues, with 57 up-regulated and 55 down-regulated genes. D. Protein-protein interaction (PPI) network analysis of the 112 differentially expressed genes. E. Functional enrichment analysis of the 112 genes, highlighting their biological roles and pathways.

A total of 655 GC signaling-related genes were retrieved from the MSigDB database (S2 Table, Fig 3A). Principal component analysis (PCA) revealed distinct expression patterns of these genes between paracarcinoma and KIRC tissues (Fig 3B). Among the 655 genes, 112 demonstrated significant differential expression between paracarcinoma and KIRC samples, with 57 genes upregulated and 55 genes downregulated (S4 Table, Fig 3C). PPI network analysis further demonstrated strong interactions among these differentially expressed genes (Fig 3D). The strength of gene interactions within this PPI network was assessed using the combined score from the STRING database, and these scores are provided in S5 Table. Functional enrichment analysis indicated that the 112 genes were predominantly involved in GC-related signaling pathways, cell metabolism (including carboxylic acid metabolism, carbon metabolism, lipid metabolism, glyoxylate and dicarboxylate metabolism, and valine, leucine and isoleucine metabolism), and cell cycle regulation (S6 Table, Fig 3E).

Using NMF clustering analysis of 112 gene expression profiles in the TCGA-KIRC cohort, we identified two distinct clusters (C1 and C2), as demonstrated by the line charts and consensus matrix. Survival analysis revealed that patients in the C2 subgroup had significantly poorer OS compared to those in C1 (*P* < 0.001). Furthermore, the C2 subgroup showed enhanced GC signaling pathway activity (*P* < 0.01, S1 Fig). Differential expression analysis between these two clusters identified 228 DEGs, comprising 167 up-regulated and 61 down-regulated genes (S7 Table, S1 Fig), from all RNA-seq detected genes in the TCGA-KIRC cohort. Functional enrichment analysis of these DEGs demonstrated their predominant involvement in immune regulation and inflammatory response pathways (S8 Table, S1 Fig). These analyses re-confirmed the tight association between GC signaling-related genes and the progression of KIRC subjects.

### Identification of prognostically significant genes within the GC signaling

**Fig 4:**
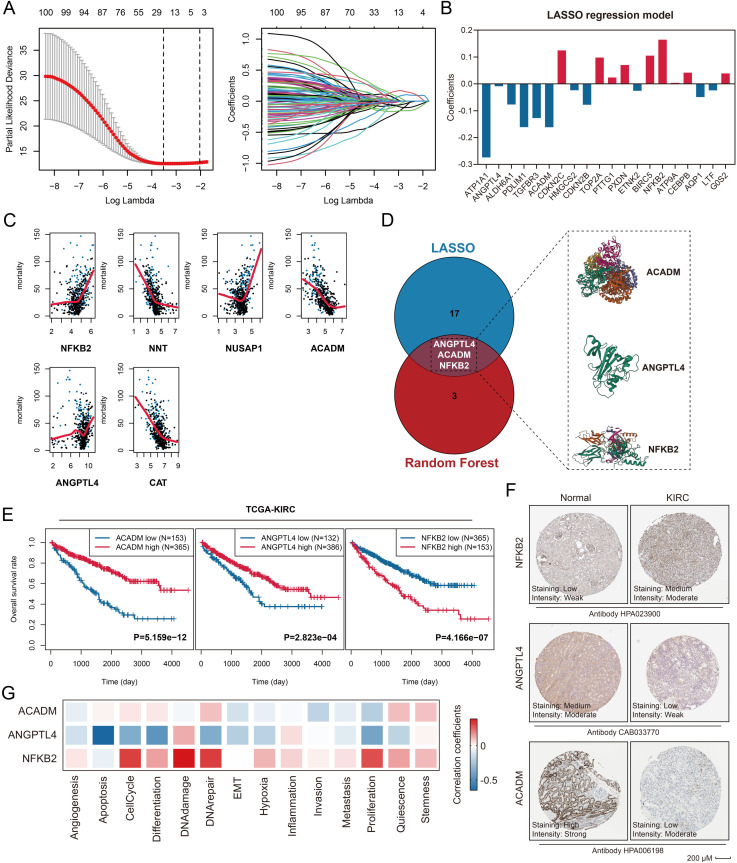
Identification of ACADM, ANGPTL4, and NFKB2 as significant predictors of OS in the TCGA-KIRC cohort. A. Twenty genes were identified as significant predictors of OS using LASSO regression with 10-fold cross-validation. B. The coefficients of the 20 genes in the LASSO regression model. C. Six genes were identified as significant predictors through random forest analysis. D. ACADM, ANGPTL4, and NFKB2 were co-identified by both LASSO regression and random forest analysis (left), along with their corresponding protein structures (right). E. High expression of ACADM and ANGPTL4 was associated with favorable OS, while high expression of NFKB2 indicated unfavorable OS in the TCGA-KIRC cohort. Optimal cut-off values were determined using X-tile software. The Optimal cut-off values for ANGPTL4, ACADM, and NFKB2 were 0.968, 0.728, and 0.776, respectively. F. Protein expression levels of ACADM, ANGPTL4, and NFKB2 in normal kidney and KIRC tissues, as obtained from the Human Protein Atlas (HPA, https://www.proteinatlas.org/). G. Association of ACADM, ANGPTL4, and NFKB2 with multiple malignant phenotypes of KIRC at the single-cell level, as analyzed using CancerSEA (http://biocc.hrbmu.edu.cn/CancerSEA/).

To identify key prognostic biomarkers among the 112 GC signaling-related genes in KIRC patients, we performed a comprehensive feature selection analysis. Using LASSO regression, we identified 20 genes significantly associated with OS (Fig 4A), with their corresponding coefficients detailed in Fig 4B and S9 Table. Parallel analysis using random forest feature selection identified 5 prognostic genes (Fig 4C). Through integration of both methods, three consensus genes—Acyl-CoA Dehydrogenase Medium Chain (ACADM), Angiopoietin Like 4 (ANGPTL4), and Nuclear Factor Kappa B Subunit 2 (NFKB2)—emerged as robust prognostic markers (Fig 4D). Survival analysis in the TCGA-KIRC cohort revealed that elevated expression of ACADM and ANGPTL4 was significantly associated with favorable OS (both **P* *< 0.001), whereas high NFKB2 expression correlated with poor prognosis (**P* *< 0.001, Fig 4E). IHC analysis confirmed differential protein expression patterns of these genes between normal kidney and KIRC tissues, which was obtained from HPA database (all *P* < 0.01, Fig 4F, S2 Fig). Single-cell RNA sequencing analysis further validated these findings, demonstrating that ACADM and ANGPTL4 expression negatively correlated with malignant phenotypes, including epithelial-mesenchymal transition (EMT), proliferation, and metastatic potential (Fig 4G). Conversely, NFKB2 expression showed positive associations with malignant phenotypes, such as cancer stemness, proliferative capacity, inflammatory response, and DNA damage repair processes (Fig 4G). These multi-omics findings provide strong evidence for the prognostic significance and functional relevance of these biomarkers in KIRC pathogenesis.

**Fig 5:**
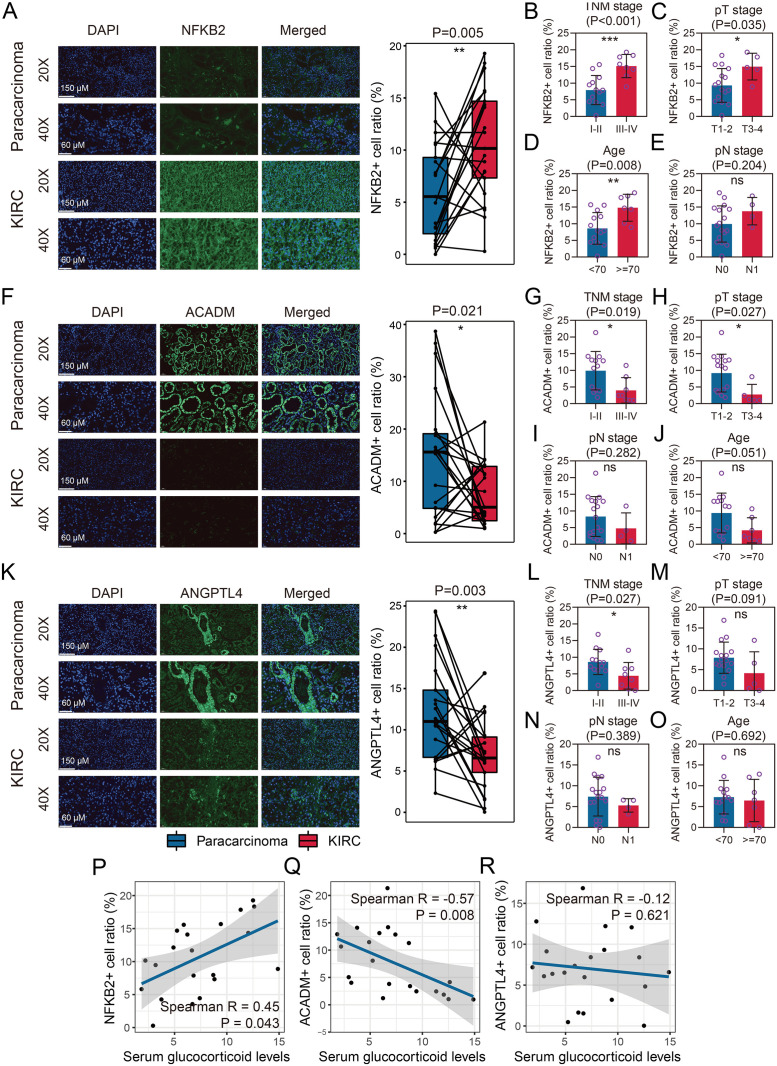
Increased NFKB2^+^ cells and decreased ACADM^+^ and ANGPTL4^+^ cells were observed in KIRC tissues. A. KIRC tissues from the local hospital showed increased NFKB2+ cells compared to paracarcinoma tissues. B-E. Association of NFKB2+ cell infiltration with TNM stages (B), pathological T stages (C), age (D), and pathological N stages (E). F. KIRC tissues from the local hospital exhibited decreased ACADM+ cells compared to paracarcinoma tissues. G-J. Association of ACADM+ cell infiltration with TNM stages (G), pathological T stages (H), pathological N stages (I), and age (J). K. KIRC tissues from the local hospital showed decreased ANGPTL4+ cells compared to paracarcinoma tissues. L-O. Association of ANGPTL4+ cell infiltration with TNM stages (L), pathological T stages (M), pathological N stages (N), and age (O). P-R. Association of the infiltration of NFKB2+ (P), ACADM+ (Q), and ANGPTL4+ (R) cells with serum glucocorticoid levels.

To validate our findings, we conducted immunofluorescence analyses using paracarcinoma and KIRC samples collected from our hospital. The results revealed a significant increase in NFKB2+ cell levels in KIRC samples compared to paracarcinoma tissues (**P* *< 0.01, Fig 5A). Further analysis indicated that NFKB2+ cell infiltration was significantly associated with TNM stages (P < 0.001, Fig 5B), pT stage (P < 0.05, Fig 5C), and age (P < 0.01, Fig 5D). However, no significant association was found with pN stage (P > 0.05, Fig 5E) or gender (P > 0.05, S3 Fig). In contrast, KIRC samples showed a marked decrease in ACADM+ cell infiltration (*P* < 0.05, Fig 5F). This reduction was more pronounced in advanced TNM stages (*P* < 0.05, Fig 5G) and pT stages (*P* < 0.05, Fig 5H), although no significant associations were observed with pN stages (*P* > 0.05, Fig 5I), age (*P* > 0.05, Fig 5J), or gender (*P* > 0.05, S3 Fig). Similarly, ANGPTL4+ cell levels were significantly reduced in KIRC samples (*P* < 0.01, Fig 5K), with a notable association with TNM stages (*P* < 0.05, Fig 5L), but not with pT stages (*P* > 0.05, Fig 5M), pN stages (*P* > 0.05, Fig 5N), age (*P* > 0.05, Fig 5O), or gender (*P* > 0.05, Fig S2C). Finally, we investigated the relationship between the identified gene expressions and serum GC levels. A positive correlation was observed between NFKB2+ cell infiltration and serum GC concentration (R = 0.45, *P* < 0.05, Fig 5P), whereas ACADM+ cell levels were negatively correlated with serum GC concentration (R = −0.57, *P* < 0.01, Fig 5Q). No significant correlation was found between ANGPTL4+ cell infiltration and serum GC levels (R = −0.12, *P* > 0.05, Fig 5R). Notably, in KIRC tissues, NFKB2+ cells, ACADM+ cells, and ANGPTL4+ cells may not be limited to tumor cells alone, but rather could represent a heterogeneous population comprising both tumor cells and various types of tumor microenvironment (TME)-infiltrating cells. The precise composition and proportional representation of these cellular subsets require further investigation.

### Pan-cancer analyses of ACADM, ANGPTL4, and NFKB2

To elucidate the prognostic implications of the identified genes, comprehensive pan-cancer analyses were performed. ACADM exhibited significant downregulation in 16 out of 32 cancer types (all *P* < 0.05), while demonstrating upregulation in glioblastoma multiforme (GBM, **P* *< 0.01, S4 Fig) when compared to adjacent normal tissues. Notably, ACADM expression emerged as a significant prognostic indicator for OS in multiple malignancies, including breast invasive carcinoma (BRCA, HR = 0.76, 95% CI = 0.60–0.98, **P* *< 0.05), brain lower grade glioma (LGG, HR = 2.51, 95% CI = 1.67–3.76, **P* *< 0.001), mesothelioma (MESO, HR = 0.55, 95% CI = 0.36–0.84, **P* *< 0.01), skin cutaneous melanoma (SKCM, HR = 0.79, 95% CI = 0.65–0.97, **P* *< 0.05), and uterine corpus endometrial carcinoma (UCEC, HR = 1.38, 95% CI = 1.06–1.81, **P* *< 0.05, S4 Fig, S10 Table).

ANGPTL4 demonstrated a distinct expression pattern, showing downregulation in 6 cancer types and upregulation in 4 malignancies (all **P* *< 0.05, S5 Fig). This gene served as a significant OS predictor across diverse cancer types, including adrenocortical carcinoma (ACC, HR = 1.50, 95% CI = 1.21–1.86, **P* *< 0.001), cervical squamous cell carcinoma and endocervical adenocarcinoma (CESC, HR = 1.20, 95% CI = 1.03–1.41, **P* *< 0.05), colon adenocarcinoma (COAD, HR = 1.31, 95% CI = 1.08–1.59, **P* *< 0.01), LGG (HR = 1.38, 95% CI = 1.17–1.63, **P* *< 0.001), lung adenocarcinoma (LUAD, HR = 1.19, 95% CI = 1.09–1.30, **P* *< 0.001), MESO (HR = 1.21, 95% CI = 1.08–1.37, **P* *< 0.01), ovarian serous cystadenocarcinoma (OV, HR = 1.18, 95% CI = 1.06–1.33, **P* *< 0.01), stomach adenocarcinoma (STAD, HR = 1.16, 95% CI = 1.01–1.34, **P* *< 0.05), uterine carcinosarcoma (UCS, HR = 1.28, 95% CI = 1.01–1.61, **P* *< 0.05), and uveal melanoma (UVM, HR = 1.83, 95% CI = 1.02–3.28, **P* *< 0.05, S5 Fig, S11 Table).

NFKB2 displayed a predominantly upregulated expression profile across 14 cancer types, with notable exceptions in kidney chromophobe (KICH, **P* *< 0.001) and pheochromocytoma and paraganglioma (PCPG, **P* *< 0.05, S6 Fig). Its expression patterns demonstrated significant prognostic value in multiple cancer types, including ACC (HR = 1.84, 95% CI = 1.06–3.19, **P* *< 0.05), BRCA (HR = 0.70, 95% CI = 0.56–0.89, **P* *< 0.01), COAD (HR = 1.47, 95% CI = 1.06–2.03, **P* *< 0.05), GBM (HR = 1.49, 95% CI = 1.05–2.11, **P* *< 0.05), kidney renal papillary cell carcinoma (KIRP, HR = 2.02, 95% CI = 1.26–3.26, **P* *< 0.01), acute myeloid leukemia (LAML, HR = 1.41, 95% CI = 1.09–1.81, **P* *< 0.01), LGG (HR = 1.39, 95% CI = 1.04–1.86, **P* *< 0.05), LUAD (HR = 1.33, 95% CI = 1.06–1.67, **P* *< 0.05), sarcoma (SARC, HR = 0.56, 95% CI = 0.41–0.77, **P* *< 0.001), SKCM (HR = 0.78, 95% CI = 0.65–0.93, **P* *< 0.01), STAD (HR = 0.75, 95% CI = 0.57–0.98, **P* *< 0.05), and thymoma (THYM, HR = 3.61, 95% CI = 1.47–8.91, **P* *< 0.01, S6 Fig, S12 Table). These findings collectively underscore the potential of these genes as valuable prognostic biomarkers across diverse cancers.

### GSRS served as a robust prognosis predictor across multiple KIRC cohorts

**Fig 6:**
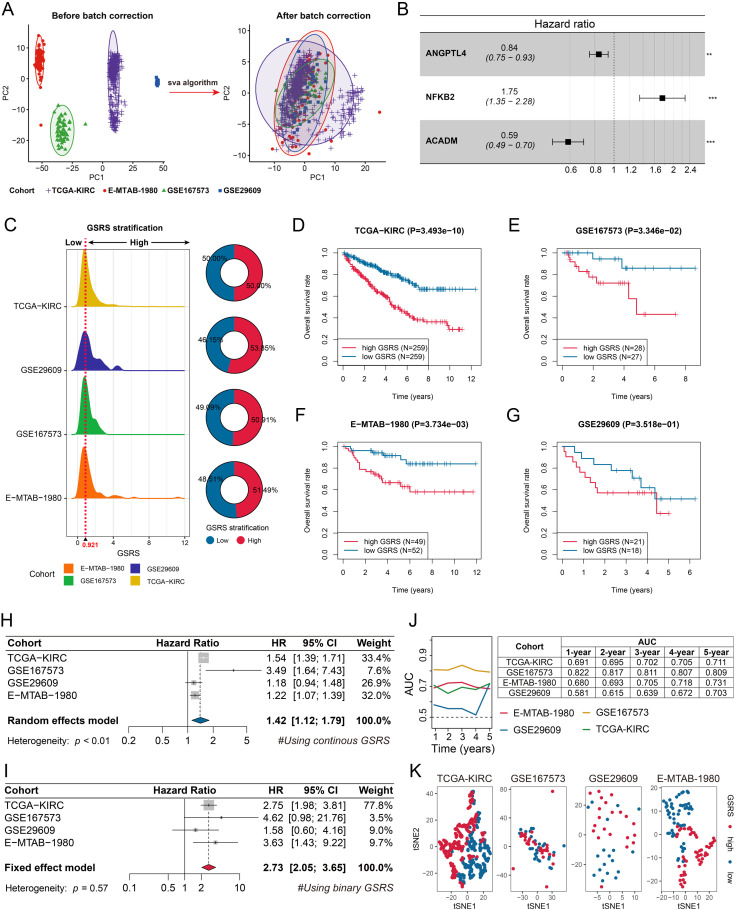
Development and validation of the GSRS. A. Batch effects across the TCGA-KIRC, E-MTAB-1980, GSE167573, and GSE29609 cohorts were minimized using the sva algorithm. B. A multivariate Cox regression model was constructed based on the expression levels of ACADM, ANGPTL4, and NFKB2, with the hazard ratios (HRs) for these genes displayed. C. The GSRS for each KIRC subject was calculated using the multivariate Cox regression model. Subjects from these KIRC cohorts were stratified into low- and high-GSRS subgroups based on the median GSRS value in the TCGA-KIRC cohort. D-G. The significant differences in OS between low- and high-GSRS subgroups in the TCGA-KIRC (D), GSE167573 (E), E-MTAB-1980 (F), and GSE29609 (G) cohorts. H-I. Meta-analyses were performed to aggregate the HRs for continuous (H) and binary (I) GSRS across the cohorts. J. Time-dependent ROC analyses evaluated the predictive performance of GSRS in the KIRC cohorts. K. Unsupervised t-distributed stochastic neighbor embedding (t-SNE) revealed distinct genomic features between low- and high-GSRS subgroups in the KIRC cohorts.

PCA was conducted to assess the effectiveness of batch effect removal in both the training (TCGA-KIRC) and validation cohorts (E-MTAB-1980, GSE29609, and GSE167573), as illustrated in Fig 6A. Utilizing the expression levels of ACADM, ANGPTL4, and NFKB2, a multivariate Cox regression model was developed (Fig 6B). The GSRS for each KIRC subject was calculated using the formula: GSRS = −0.177*exp(ANGPTL4) + 0.560*exp(NFKB2) – 0.532*exp(ACADM). Subjects were then stratified into low- and high-GSRS subgroups based on the median GSRS value (0.921) from the TCGA-KIRC cohort, as shown in Fig 6C.

Analysis revealed that high-GSRS cases in the TCGA-KIRC cohort (**P* *< 0.001, Fig 6D), GSE167573 cohort (**P* *< 0.05, Fig 6E), and E-MTAB-1980 cohort (**P* *< 0.001, Fig 6F) were associated with significantly poorer OS. However, the GSE29609 cohort did not show a significant OS difference between low- and high-GSRS cases (**P* *> 0.05, Fig 6G), potentially due to its limited sample size and the inherent heterogeneity of KIRC. To address this, meta-analyses were performed, which confirmed GSRS as a significant predictor of OS, both as a continuous variable (pooled HR = 1.42, 95% CI = 1.12–1.79, Fig 6H) and as a binary variable (pooled HR = 2.73, 95% CI = 2.05–3.65, Fig 6I).

Further validation through time-dependent ROC analyses demonstrated that the AUCs for GSRS exceeded 0.5 across all KIRC cohorts, underscoring the robustness of GSRS as a prognostic tool (Fig 6J). Additionally, t-distributed stochastic neighbor embedding (t-SNE) analysis revealed distinct genomic features between high- and low-GSRS subjects, further corroborating our findings (Fig 6K). In t-SNE analysis, all genes were included in the analysis and not limited to a specific gene set.

Notably, in the TFE3 translocation-subtype cohort (GSE167573), continuous GSRS showed robust prognostic performance with the highest AUC in time-dependent ROC analysis (Fig 6J), while the dichotomized GSRS (based on the median cut-off from TCGA-KIRC) demonstrated reduced but still significant prognostic power in Kaplan-Meier analysis (Fig 6E). Further supporting this, t-SNE revealed incomplete separation between high- and low-risk groups (Fig 6K), and meta-analyses indicated that GSE167573 carried the smallest weight with the widest 95% CI for both continuous and dichotomized GSRS (Fig 6H-I). These findings suggest that although continuous GSRS remains an effective prognostic tool in TFE3-translocated KIRC, the generalized dichotomization approach may be suboptimal owing to its unique biology. Future studies should seek to validate and optimize subtype-specific GSRS thresholds to improve prognostic accuracy.

The GSE167573 cohort represents the TFE3 translocation subtype of KIRC. Although TFE3-translocated KIRC is one of the molecular subtypes of KIRC, including this cohort may potentially introduce bias into the analysis. Therefore, we performed a repeated meta-analysis after excluding the GSE167573 cohort. The results demonstrated that even after its exclusion, GSRS remained a significant prognostic marker for poor outcomes in KIRC patients, both as a continuous variable (HR = 1.32, 95% CI = 1.11–1.58) and as a binary variable (HR = 2.68, 95% CI = 2.00–3.60, S7 Fig).

**Fig 7:**
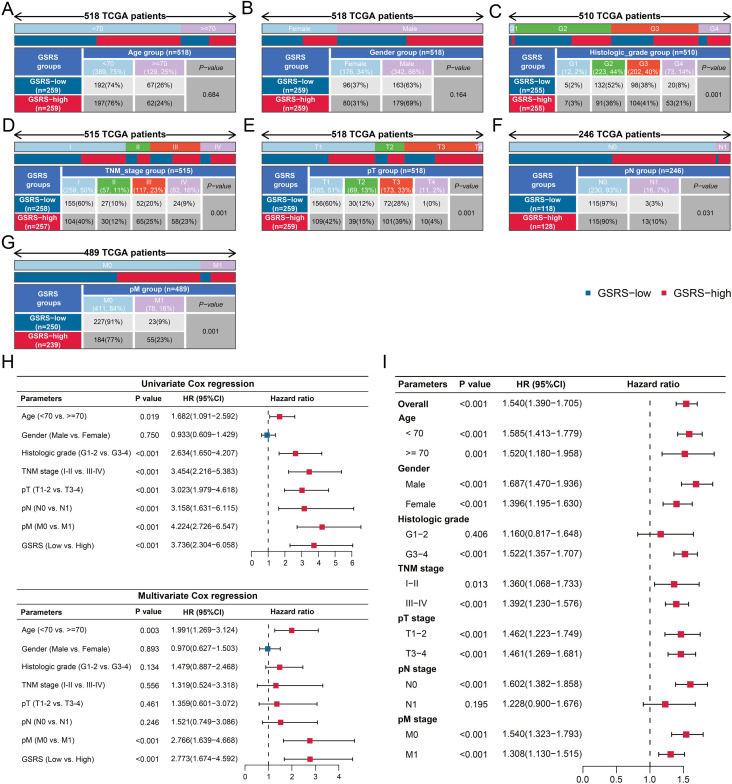
Clinicopathological associations of GSRS. A-G. Associations of GSRS stratification with age (A), gender (B), histological grade (C), TNM stages (D), pathological T stages (E), pathological N stages (F), and pathological M stages (G). H. Univariate (top) and multivariate (bottom) Cox regression analyses demonstrating that GSRS serves as an independent predictor of OS after converting all continuous variables into binary variables. I. Subgroup analyses evaluating the predictive performance of GSRS for OS.

The clinicopathological characteristics of GSRS were comprehensively analyzed. We utilized batch-corrected transcriptome sequencing data for our analysis, as batch correction has been demonstrated to significantly enhance comparability across different groups and mitigate substantial noise introduced by batch effects. Our findings revealed no significant correlation between GSRS stratification and age (**P* *> 0.05, Fig 7A) or gender (**P* *> 0.05, Fig 7B). However, GSRS demonstrated significant associations with several key pathological parameters, including histological grade (**P* *< 0.01, Fig 7C), TNM stages (**P* *< 0.01, Fig 7D), pT stages (**P* *< 0.01, Fig 7E), pN stages (**P* *< 0.05, Fig 7F), and pathological M (pM) stages (**P* *< 0.01, Fig 7G). Following the transformation of all clinicopathological features into binary variables and exclusion of cases with missing values, our analysis demonstrated that GSRS emerged as an independent predictor of OS. This was evident in both univariate (HR = 3.74, 95% CI = 2.30–6.06, **P* *< 0.001) and multivariate Cox regression analyses (HR = 2.77, 95% CI = 1.67–4.59, **P* *< 0.001, Fig 7H). Subgroup analyses further confirmed the prognostic value of GSRS across most subgroups, with the exception of G1-2 (HR = 1.16, 95% CI = 0.82–1.65, **P* *> 0.05) and pN1 (HR = 1.23, 95% CI = 0.90–1.68, **P* *> 0.05, Fig 7I) subgroups, where statistical significance was not achieved. The forest plot was used to visualize the subgroup analysis results (Fig 7I). Notably, the GSRS exhibits comparable prognostic value in KIRC patients both below and above 70 years of age, indicating that GC-driven KIRC progression is largely age-independent. However, in our institutional cohort, serum GC levels correlated positively with age (Fig 2E). This discrepancy may stem from the limited sample size of our cohort, warranting further investigation into the relationship between age and GC levels in KIRC.

We further investigated the relationship between GSRS stratification and the clustering patterns derived from 112 GC signaling-related genes. Our analysis revealed that KIRC cases classified within C2 demonstrated significantly elevated GSRS levels compared to C1 (**P* *< 0.001, S8 Fig). Moreover, a significant positive association was observed between GSRS and the activity of GC signaling pathways (**P* *< 0.001, S9 Fig).

HSD11B1 and GR are key components of GC signaling. We next evaluated whether combining GSRS with GR and/or HSD11B1 could improve prognostic prediction for KIRC patients. Analyses using the TCGA-KIRC cohort and HPA database showed that expression levels of GR and HSD11B1 were not significantly associated with patient OS (both *P* > 0.05, S10 Fig) and were only moderately elevated in KIRC compared to normal renal tissues (S10 Fig). Multivariate Cox regression models integrating GSRS with GR and/or HSD11B1 did not significantly enhance prognostic accuracy compared to GSRS alone and, in fact, exhibited poorer performance in predicting OS at years two and four (S10 Fig).

### Pan-cancer analyses of GSRS

As this study represents the first establishment of a GC signaling-related risk signature in malignancies, we proceeded to evaluate the predictive performance of GSRS across 32 additional cancer types. Our analysis revealed significantly elevated GSRS levels in tumor samples compared to adjacent normal tissues in 16 out of 33 cancer types, including KIRC (all **P* *< 0.01, S11 Fig). Univariate Cox regression analysis demonstrated that GSRS serves as a significant prognostic indicator for OS in multiple cancer types: KIRP (HR = 1.78, 95% CI = 1.12–2.83, **P* *< 0.05), LAML (HR = 1.40, 95% CI = 1.01–1.92, **P* *< 0.05), LGG (HR = 0.51, 95% CI = 0.31–0.82, **P* *< 0.01), and SARC (HR = 0.51, 95% CI = 0.35–0.74, **P* *< 0.001, S11 Fig, S13 Table). These findings collectively suggest that GSRS is strongly associated with tumorigenesis at a pan-cancer level and represents a valuable prognostic biomarker, particularly in KIRP and LAML.

### GSRS was positively associated with CD8^+^ T cells infiltration in KIRC tissues

**Fig 8:**
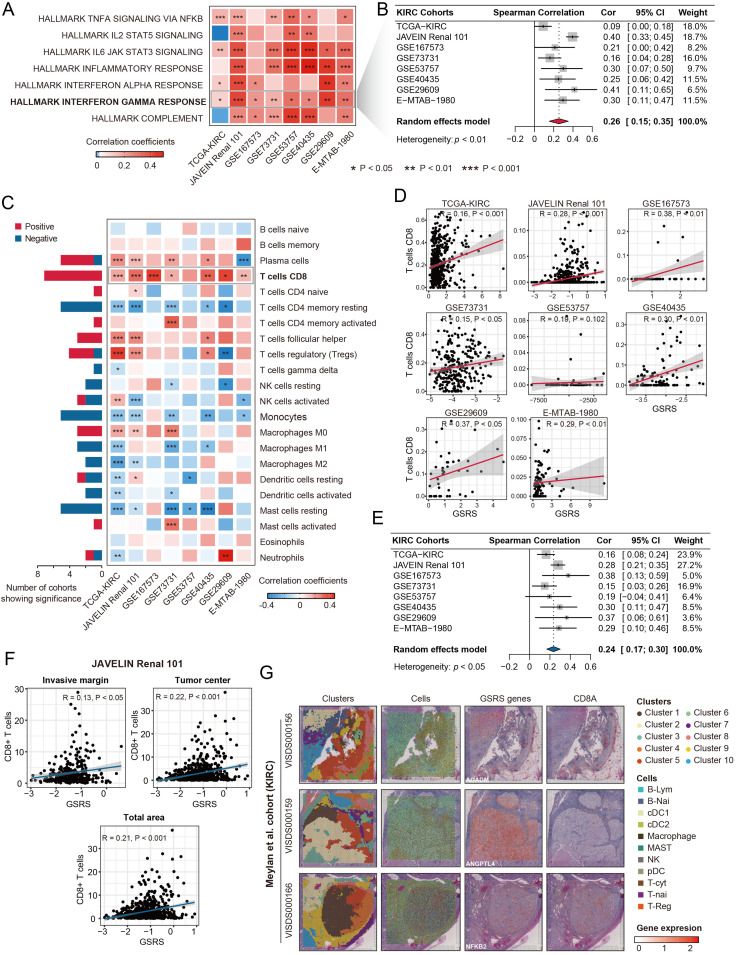
GSRS was positively associated with CD8^+^ T cell infiltration levels in KIRC tissues. A. Association of GSRS with the response levels of immune-related pathways across multiple KIRC cohorts. B. Meta-analysis revealed a positive association between GSRS and interferon-γ response levels. C. Association of GSRS with immune cell infiltration levels, highlighting a robust correlation with CD8+ T cells infiltration. D. Correlation coefficients of GSRS and CD8+ T cell infiltration across KIRC cohorts. E. Meta-analysis confirms a significant positive association between GSRS and CD8+ T cell infiltration levels. F. Association of GSRS with CD8+ T cell infiltration in the invasive margin, tumor center, and all areas in KIRC samples from the JAVELIN Renal 101 cohort. G. Spatial transcriptomics analysis demonstrates the association of ACADM, ANGPTL4, and NFKB2 with CD8A, as obtained from the CROST database (https://ngdc.cncb.ac.cn/crost/home).

Given the well-established link between GC signaling and anti-tumor immune responses, we proceeded to investigate the immunogenetic characteristics of the GSRS. Our initial analysis focused on the relationship between GSRS and the activity of various immune-related pathways. Across eight independent KIRC cohorts, we identified a significant positive correlation between GSRS and interferon-γ (IFN-γ) response (all **P* *< 0.05, S14 Table, Fig 8A). The pooled correlation coefficient of 0.26 (95% CI = 0.15–0.35) further substantiated this robust association (Fig 8B). IFNγ, a pivotal cytokine primarily secreted by activated T cells, including Th1 and cytotoxic T cells, and natural killer (NK) cells, plays a crucial role in immune regulation [28]. Given the synergistic effects of IFNγ and TNFα on NF-κB signaling activated by GCs [29], we further conducted a meta-analysis to assess the relationship between GSRS and TNFα signaling. The results demonstrated a significant positive association between GSRS and TNFα signaling activity across eight independent KIRC cohorts (pooled R = 0.18, 95% CI: 0.14–0.22, S12 Fig).

Subsequent analysis using the CIBERSORT-ABS algorithm revealed significant associations between GSRS and immune cell infiltration patterns (Fig 8C). Specifically, GSRS demonstrated consistent positive correlations with CD8+ T cell infiltration levels across multiple cohorts: TCGA-KIRC (R = 0.16, **P* *< 0.001), JAVELIN Renal 101 (R = 0.28, **P* *< 0.001), GSE167573 (R = 0.38, **P* *< 0.01), GSE73731 (R = 0.15, **P* *< 0.05), GSE40435 (R = 0.30, **P* *< 0.01), GSE29609 (R = 0.37, **P* *< 0.05), and E-MTAB-1980 (R = 0.29, **P* *< 0.01). However, no significant association was observed in the GSE53757 cohort (R = 0.19, **P* *> 0.05, Fig 8D). Meta-analysis of these cohorts confirmed a significant overall association between GSRS and CD8+ T cell infiltration (pooled R = 0.24, 95% CI = 0.17–0.30, Fig 8E).

These findings were further corroborated by multiple deconvolution algorithms: TIMER (R = −0.01, **P* *> 0.05), CIBERSORT (R = 0.15, **P* *< 0.001), QUANTISEQ (R = 0.20, **P* *< 0.001), MCP-COUNTER (R = 0.11, **P* *< 0.05), and xCELL (R = 0.18, **P* *< 0.001, S13 Fig, S15 Table). Clinical validation in the JAVELIN Renal 101 cohort, where Motzer et al. employed IHC to quantify CD8+ T cell infiltration in tumor invasive margins, centers, and total areas, revealed significant positive correlations between GSRS and CD8+ T cell infiltration in all three regions: invasive margin (R = 0.13, **P* *< 0.05), tumor center (R = 0.22, **P* *< 0.001), and total areas (R = 0.21, **P* *< 0.001, Fig 8F).

CD8A encodes the α-chain of the CD8 co-receptor protein, and its expression levels directly correlating with CD8 protein abundance on cell surfaces [30]. Spatial transcriptomic analysis provided additional insights, revealing spatial exclusivity between ACADM/ANGPTL4 and CD8A, while demonstrating co-localization of NFKB2 and CD8A in KIRC samples (Fig 8G). These findings collectively highlight the intricate relationship between GSRS and CD8+ T cell-mediated immune responses in KIRC.

To further investigate the pan-cancer relevance of GSRS, we systematically evaluated its association with CD8+ T cell infiltration across 32 different cancer types. Remarkably, our analysis revealed a statistically significant positive correlation between GSRS and CD8+ T cell infiltration in 23 out of 32 cancer types (all **P* *< 0.05, S16 Table), demonstrating a widespread association of this molecular signature with CD8+ T cell infiltration across diverse malignancies.

### Patients with high GSRS demonstrated elevated levels of TEX and showed reduced responsiveness to immunotherapy

**Fig 9:**
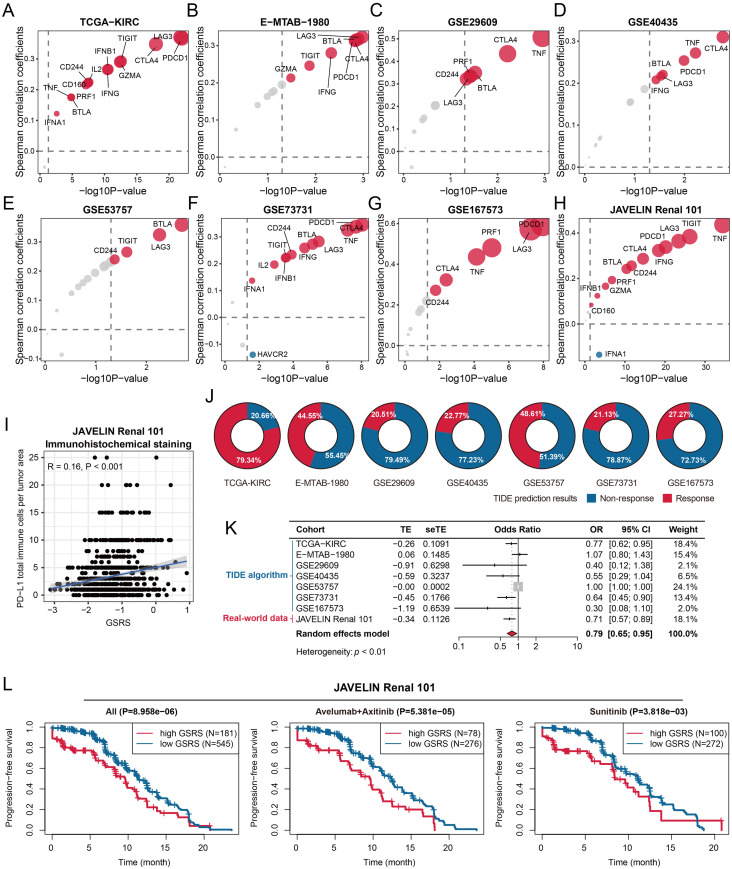
GSRS was positively associated with T cell exhaustion (TEX) and predicted a reduced likelihood of response to immunotherapy. A-H. Association between GSRS and TEX-related genes in the TCGA-KIRC (A), E-MTAB-1980 (B), GSE29609 (C), GSE40435 (D), GSE53757 (E), GSE73731 (F), GSE167573 (G), and JAVELIN Renal 101 (H) cohorts. I. Positive association between GSRS and PD-L1 protein expression levels in KIRC samples from the JAVELIN Renal 101 cohort. J. Evaluation of the likelihood of immunotherapy response in KIRC cohorts using the tumor immune dysfunction and exclusion (TIDE) algorithm. K. Meta-analysis demonstrates that KIRC patients with high GSRS are less likely to benefit from immunotherapy. L. Unfavorable progression-free survival in KIRC patients with high GSRS across all subjects (left), subjects treated with Avelumab in combination with Axitinib (middle), and subjects receiving Sunitinib monotherapy (right) within the JAVELIN Renal 101 cohort. The optimal cut-off values were detected by X-tile software.

CD8+ T cells, known for their cytotoxic function in anti-tumor immunity, play a crucial role in identifying and destroying tumor cells. Our research reveals a significant positive correlation between GSRS levels and CD8+ T cell infiltration in KIRC tissues, with higher GSRS levels associated with a poorer prognosis. This led us to hypothesize that CD8+ T cells in KIRC tissues with increased GSRS levels are in an enhanced state of TEX. To test this, we first examined the relationship between GSRS and TEX marker gene expressions. Our analysis across multiple cohorts—TCGA-KIRC (Fig 9A), E-MTAB-1980 (Fig 9B), GSE29609 (Fig 9C), GSE40435 (Fig 9D), GSE53757 (Fig 9E), GSE73731 (Fig 9F), GSE167573 (Fig 9G), and JAVELIN Renal 101 (Fig 9H, S17 Table)—showed a significant positive association between GSRS and TEX feature genes. Further clinical validation in the JAVELIN Renal 101 cohort confirmed a significant correlation between PD-L1 protein expressions and GSRS (R = 0.16, **P* *< 0.001, Fig 9I), strongly supporting our hypothesis. It is noteworthy that in the JAVELIN Renal 101 cohort, PD-L1 protein expression levels were assessed prior to treatment [19]. To further support our findings, we assessed GSRS and TEX co-expression in KIRC using spatial transcriptomics, which also revealed a positive association (S14 Fig).

TEX is recognized as a critical factor in the failure of ICIs treatment [31]. Leveraging this insight, we assessed the predictive value of GSRS for immunotherapy efficacy. Using the TIDE algorithm, we evaluated the response rates to ICIs in KIRC cohorts (Fig 9J). Combining TIDE analysis with therapeutic response data from the JAVELIN Renal 101 cohort treated with avelumab plus axitinib, GSRS demonstrated significant predictive performance for immunotherapy (pooled OR = 0.79, 95% CI = 0.65–0.95, Fig 9K). Moreover, high GSRS levels were associated with unfavorable PFI across all subjects (**P* *< 0.001), those treated with avelumab plus axitinib (**P* *< 0.001), and cases treated with sunitinib (**P* *< 0.001, Fig 9L). Optimal cut-off values were determined using the X-tile method.

We also compared the predictive ability of GSRS with commonly-used predictors. Univariate logistic regression indicated that GSRS was a significant predictor of immunotherapy response (OR = 0.70, 95% CI = 0.52–0.93, **P* *< 0.05), outperforming tumor mutational burden (TMB, OR = 0.92, 95% CI = 0.74–1.12, **P* *> 0.05) and microsatellite instability (MSI, OR = 0.01, 95% CI = 0.00–9.66, **P* *> 0.05, S15 Fig) in the TCGA-KIRC cohort. Compared to immune checkpoint genes, GSRS showed the highest HR (HR = 1.83, 95% CI = 1.33–2.52, **P* *< 0.001, S16 Fig) in KIRC subjects receiving avelumab plus axitinib treatment in the JAVELIN Renal 101 cohort. Overall, GSRS emerges as a promising clinical biomarker for predicting immunotherapeutic response in KIRC.

### Identifying the candidate chemotherapeutic agents effective for high-GSRS cases

Patients with high GSRS exhibited poor prognosis and demonstrated limited responsiveness to immunotherapy. This underscores the clinical significance of identifying effective chemotherapeutic agents for high-GSRS KIRC patients. Using the oncoPredict R package, we systematically evaluated the IC50 values of 198 chemotherapeutic agents in the TCGA-KIRC cohort (S18 Table). Subsequent Wilcoxon signed-rank tests identified the top 20 chemotherapeutic agents showing the most pronounced sensitivity differences between high- and low-GSRS groups (S19 Table). Notably, high-GSRS patients showed enhanced sensitivity to several agents out of the top 20 drugs, including ULK1_1989, Mirin, XAV939, Vinorelbine, Epirubicin, GNE-317, Topotecan, Irinotecan, Camptothecin, Elephantin, AZD7762, MG-132, Palbociclib, 5-Fluorouracil, Vinblastine, Rapamycin, Teniposide, Pictilisib, and PRIMA-1MET (S17 Fig). Concurrently, it was found that high GSRS was associated with reduced sensitivity to Axitinib (S18 Fig). These findings not only provide valuable insights into the molecular characteristics of high-GSRS KIRC patients but also offer a clinically relevant therapeutic roadmap for this challenging patient population. The identified drug candidates warrant further investigation through prospective clinical trials to validate their therapeutic potential in high-GSRS KIRC patients.

### Knockdown of Nfkb2 inhibited tumor growth and TEX state *in vivo*

The GSRS was composed of three key genes: ACADM, ANGPTL4, and NFKB2. While the roles of ACADM and ANGPTL4 in KIRC have been well-documented in previous studies [32,33], we focused our investigation on NFKB2 for experimental validation. Spatial transcriptomics analysis revealed that although NFKB2 did not exhibit significant co-expression with GR in KIRC tissues, it was co-expressed with HSD11B1, further confirming a significant association between NFKB2 and GC signaling in KIRC (S19 Fig).

**Fig 10:**
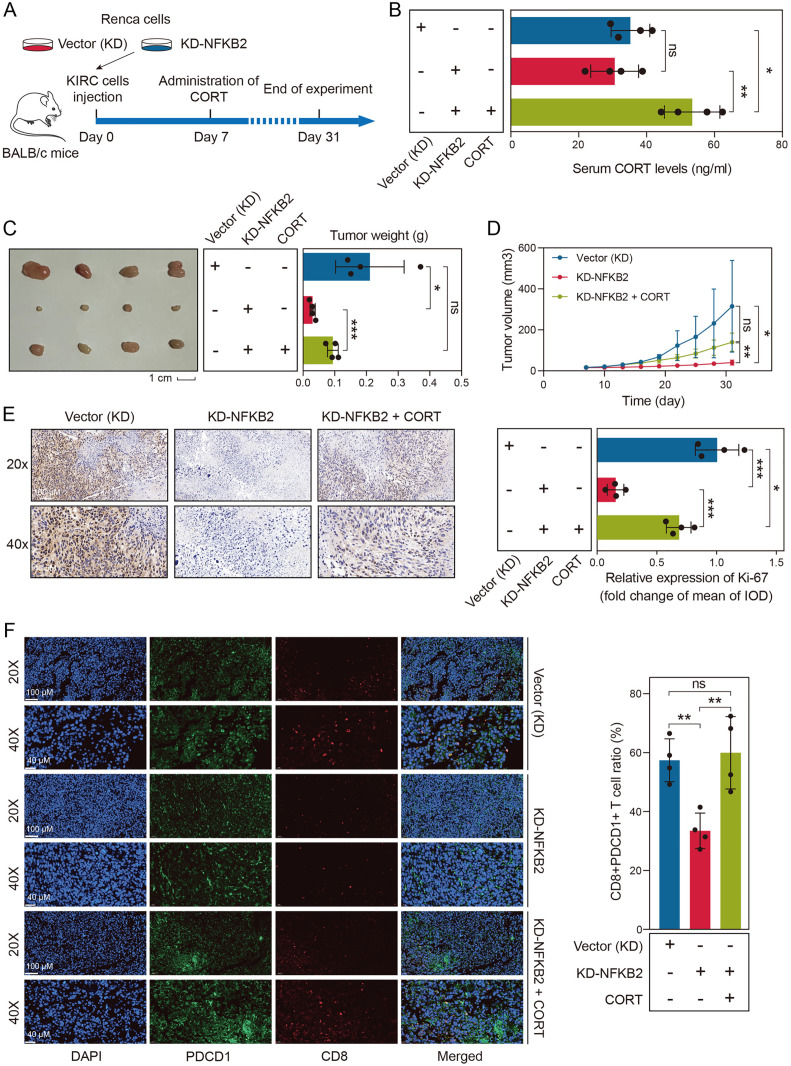
Knockdown of Nfkb2 inhibited tumor growth and TEX levels *in vivo.* A. Workflow of the *in vivo* experiments. On day 0, renal cells with or without Nfkb2 knockdown were injected subcutaneously into BALB/c mice. On day 7, mice injected with Nfkb2-knockdown renal cells were treated with CORT. B. Serum CORT levels in BALB/c mice. C. Knockdown of Nfkb2 inhibited tumor growth, an effect that was reversed by CORT treatment. D. Tumor size in mice during the *in vivo* experiment. E. Knockdown of Nfkb2 reduced Ki67 expression, an effect that was reversed by CORT treatment. F. Knockdown of Nfkb2 suppressed the infiltration of CD8+PDCD1+ T cells in tumors, but CORT treatment reversed this effect.

To elucidate its functional significance, we established a Renca cell line with stable Nfkb2 knockdown, with knockdown efficiency confirmed by RT-qPCR analysis (both **P* *< 0.01, S20 Fig). In our *in vivo* experiments, we subcutaneously injected Renca cells (both control and Nfkb2-knockdown) into BALB/c mice. Starting from day 7 post-injection, the CORT subgroup received CORT treatment (Fig 10A). After 31 days, we collected peripheral blood samples via orbital sinus puncture for serum CORT quantification. The CORT group showed significantly elevated serum CORT levels (both **P* *< 0.05), while no significant differences were observed between the Vector (KD) and KD-NFKB2 groups (**P* *> 0.05, Fig 10B). Importantly, Nfkb2 knockdown significantly suppressed tumor growth (**P* *< 0.05), an effect that was subsequently reversed by CORT supplementation (**P* *< 0.001, Fig 10C-D). The tumor volume of the mice in each group is provided in S20 Table. Furthermore, Nfkb2 knockdown markedly reduced Ki67 expression (**P* *< 0.001), which was also counteracted by CORT administration (**P* *< 0.001, Fig 10E).

Through IHC analysis, we made an intriguing observation: CORT supplementation upregulated Nfkb2 protein expression in tumor tissues (**P* *< 0.01, S21 Fig), suggesting that GCs may enhance Nfkb2 expression in KIRC. The upregulation of Nfkb2 expression by GCs may be mediated by direct transcriptional activation or enhanced protein stability, and the exact mechanism requires further validation through subsequent molecular biology experiments.

Our analyses revealed a strong correlation between GSRS and TEX states. To further investigate this relationship, we examined the infiltration levels of CD8+PDCD1+ T cells across different experimental groups. The results demonstrated that Nfkb2 knockdown significantly reduced CD8+PDCD1+ T cell infiltration (**P* *< 0.01), while CORT supplementation promoted their infiltration (**P* *< 0.01, Fig 10F). These experimental results provide robust validation of our bioinformatics findings. However, the precise biological mechanisms underlying these associations remain to be fully elucidated and warrant further investigation in subsequent studies. Future studies should investigate the effects of CORT and/or GR inhibitors in NFKB2-knockdown KIRC cells *in vivo* to determine whether NFKB2’s pro-tumorigenic functions depend on GC signaling and GR activity.

### Comparison of GSRS with the established models

To comprehensively evaluate the performance of the GSRS model, we compared it with 44 previously published prognostic models incorporating the same three genes, obtained from PubMed (Accessed date: 13 September 2025). These models were associated with diverse biological processes including metabolism, immunity, hypoxia, senescence, and the cGAS-STING pathway (S21 Table). The GSRS exhibited robust prognostic performance, ranking fifth in predicting 5-year OS and third for 1-, 2-, and 4-year OS. Notably, it significantly outperformed all other models in predicting 3-year OS (S22 Fig), underscoring its superior predictive capability.

## Discussion

Emerging evidence has increasingly demonstrated that GCs play a pivotal role in tumor initiation and progression through their regulatory effects on anti-tumor immune responses and the promotion of malignant phenotypes in tumor cells [26,34,35]. Although GCs have been demonstrated to promote apoptosis in tumor cells of lymphoma and leukemia [8], they can facilitate tumor progression in solid tumors through various mechanisms. GCs promote metastasis in colorectal, pancreatic, and prostate cancers by directly regulating targets such as the GR, TET2, and microRNA-378 [36–38]. In murine models of renal cancer, endogenous GCs have been demonstrated to impair dendritic cell maturation and antigen-presenting capacity, while also suppressing T cell-mediated cytotoxicity, thereby curtailing the efficacy of both immune checkpoint inhibitors and Toll-like receptor agonists [35]. Despite these significant findings, the specific roles and mechanisms of GCs in KIRC remain poorly understood. To address this knowledge gap, we conducted a case-control study with a 1:2 ratio (KIRC patients to healthy donors) to investigate serum GC level differences in our local patient population. Using a standardized ELISA protocol, we quantitatively analyzed serum GC concentrations in both KIRC patients and healthy controls. Our findings revealed a significant elevation in serum GC levels among KIRC patients compared to healthy donors. Furthermore, we observed a positive correlation between serum GC concentrations and tumor malignancy grade in KIRC patients. These results not only support the involvement of GCs in KIRC pathogenesis but also suggest GC signaling-related genes as potential biomarkers for tumor progression.

However, the source of abnormally elevated GC levels in the serum of these KIRC patients warrants further investigation. Although the hypothalamic-pituitary-adrenal (HPA) axis is the primary regulator of systemic GCs, local production and metabolism within the TME significantly influence GC levels. Tumor cells themselves, as well as stromal cells like fibroblasts or adipocytes, can express enzymes involved in GC synthesis (e.g., 11β-hydroxysteroid dehydrogenase type 1, HSD11B1) or inactivation (e.g., 11β-hydroxysteroid dehydrogenase type 2, HSD11B2; Cytochrome P450 3A4/5, CYP3A4/5). Dysregulation of these enzymes, particularly overexpression of HSD11B1, which regenerates active cortisol from inactive cortisone, can lead to elevated GC concentrations within tumors, independent of systemic HPA axis activity [39]. Future studies should specifically examine the expression profiles of GC synthesis (e.g., CYP11B1) and metabolism enzymes (especially HSD11B1, HSD11B2, CYP3A4/5) within KIRC tumors and correlate them with serum GC levels to elucidate the relative contributions of systemic versus local GC sources in this malignancy.

This study provides a preliminary assessment of the diagnostic potential of serum GC in KIRC. The results indicate that serum GC displays high sensitivity and specificity in distinguishing KIRC cases. Given its established use in endocrinology, serum GC testing could be adapted as an adjunctive method for KIRC diagnosis. Several practical aspects, however, require consideration. The ELISA method used here costs approximately $20–50 per test, proving more affordable and operationally simpler than techniques like liquid chromatography-tandem mass spectrometry (LC-MS/MS). Nonetheless, variability among commercial ELISA kits poses standardization challenges. Regarding turnaround time, the assay required 4–6 hours from sampling to results, aligning with routine clinical timelines and enabling same-day diagnosis. Further investigation should explore combining serum GC with other biomarkers, such as ACADM, ANGPTL4, and NFKB2, to improve diagnostic accuracy for KIRC.

The identification of robust and accurate prognostic biomarkers has consistently been a focal point in KIRC research [40–42]. Recent advancements have demonstrated that integrating genomic features into prognostic predictive models significantly enhances KIRC prognosis prediction. Notably, analyses focusing on specific biological processes, such as cuproptosis [43], disulfidptosis [44], and lactate metabolism [45], have yielded substantial improvements in prognostic accuracy. However, despite these advancements, no gene signature related to GC signaling has been established as a prognostic indicator in malignancies, particularly in KIRC. In this study, we initiated our investigation by compiling GC signaling-related genes from the MSigDB database. We utilized the TCGA-KIRC cohort as training dataset and incorporated seven independent KIRC cohorts from GEO, ArrayExpress, and previous publications as validation sets. Through feature selection employing LASSO and random forest analyses, we identified ACADM, ANGPTL4, and NFKB2 as significant genes associated with OS in KIRC patients. These genes were subsequently integrated into a multivariate Cox regression model to calculate a GSRS for each KIRC subject. Analysis of clinical samples from our local hospital revealed that KIRC samples exhibited elevated levels of NFKB2+ cells and reduced levels of ACADM+ and ANGPTL4+ cells. Meta-analyses across multiple KIRC cohorts confirmed that GSRS serves as a significant prognostic indicator. Importantly, we found that GSRS positively correlated with CD8+ T cell infiltration and increased TEX states, which likely contribute to the poor prognosis observed in high-GSRS subjects. Given that increased TEX is a critical factor in the failure of ICIs treatments [46], we further assessed the predictive capability of GSRS for immunotherapeutic response using both computational methods and clinical data. Owing to tumor heterogeneity and variations in clinical baseline characteristics, GSRS was predictive of immunotherapy outcomes in only three KIRC cohorts. However, a meta-analysis revealed that subjects with a high GSRS were significantly less likely to benefit from immunotherapy. The in vivo experiments indicated that the knockdown of Nfkb2 inhibited tumor growth and TEX states, effects reversed by the supplement of CORT, which re-confirmed our finding. Overall, GSRS served as a reliable tool for prognostic assessment and therapeutic decision-making in KIRC.

In this study, we identified three GC signaling-related genes – ACADM, ANGPTL4, and NFKB2 – as potential prognostic biomarkers for KIRC. Among these, ACADM encodes acyl-CoA dehydrogenase medium chain, a mitochondrial enzyme essential for fatty acid β-oxidation. Emerging evidence suggests that dysregulation of ACADM expression or function may contribute to metabolic reprogramming, a critical hallmark of cancer progression [47]. Consistent with this notion, multiple studies have established ACADM as a prognostic indicator in various malignancies, including KIRC [32,48–50]. Notably, GCs have been shown to downregulate fatty acid oxidation (FAO)-related genes, including ACADM, in brown adipose tissue [51]. Given the pivotal role of FAO in energy production and biosynthesis, which directly impacts tumor growth, survival, and metastatic potential, understanding the precise biological functions of ACADM and its regulatory relationship with GC signaling is of particular importance in KIRC pathogenesis. However, the mechanistic details of this relationship remain to be fully elucidated, particularly whether GC can regulate ACADM through GR, warranting further investigation to clarify the role of ACADM in GC-mediated metabolic regulation within the KIRC context.

ANGPTL4 encodes a glycosylated, secreted protein characterized by a C-terminal fibrinogen domain, which functions as an inhibitor of lipoprotein lipase, thereby playing a crucial role in regulating triglyceride metabolism and energy homeostasis. Previous studies have demonstrated that GCs regulate the expression of ANGPTL4 through the GR [52]. Additionally, ANGPTL4 has been implicated in various cancer-related processes, including tumor growth, metastasis, and angiogenesis [33]. Interestingly, both GCs and ANGPTL4 exhibit context-dependent dual roles in tumorigenesis, either promoting or suppressing cancer progression, depending on tumor type and specific alterations in the TME. Notably, the regulation of ANGPTL4 by GCs may contribute to their paradoxical effects in cancer, suggesting that ANGPTL4 could serve as a key mediator through which GCs exert either pro-tumorigenic or anti-tumorigenic actions [8–10,53]. In KIRC, ANGPTL4 has emerged as a potential prognostic marker based on analyses of public databases [33]. In this study, we provided the first clinical evidence by comparing ANGPTL4 expression levels between paracarcinoma and KIRC tissues, offering novel insights into its role in KIRC development and progression. Future research should focus on the biological functions of ANGPTL4 in KIRC, particularly in elucidating the regulatory mechanisms between glucocorticoids and ANGPTL4 within the KIRC.

The NFKB2 gene encodes a member of the nuclear factor kappa B (NF-κB) family of transcription factors, which play a crucial role in regulating anti-tumor immune response and cancer cell survival [54]. NFKB2 has been implicated in the development and progression of various cancers [55]; however, this study is the first to report its significant upregulation in KIRC, which was validated through public KIRC cohorts and local samples. Dysregulation of NFKB2 has been demonstrated to impair normal T cell receptor (TCR) signaling, ultimately resulting in TEX [56], which is consistent with our findings. In immune cells, the inhibitory effect of GCs on NF-κB signaling has been extensively documented. The canonical mechanism involves the GC-activated GR directly interfering with NF-κB transcriptional activity. GCs have also been shown to activate NF-κB signaling in stromal cells, a process that requires the synergistic action of IFNγ and TNFα [29]. Conversely, excessive activation of NF-κB can inhibit the transcriptional activation function of the GR. Specifically, activated NF-κB, particularly the RelA/p65 subunit, directly interacts with GR through protein-protein interactions, leading to the formation of a transcriptionally inactive complex. This interaction disrupts the binding of NF-κB to the DNA response elements of its target genes [57]. Furthermore, there is evidence for reciprocal regulation of their nuclear translocation. GR can sequester NF-κB subunits in the cytoplasm, while NF-κB activity can influence GR nuclear residency [58]. The precise feedback mechanisms governing GR and NF-κB complex nuclear translocation and expression specifically in KIRC cells in response to dynamic GC concentration changes warrant further detailed investigation. In this study, we observed that supplementation with CORT led to an upregulation of Nfkb2 expression in subcutaneous tumor tissues of mice. This finding suggests that GCs can activate NF-κB signaling in KIRC tissues under in *vivo* conditions. However, the precise mechanisms underlying this activation, particularly whether it depends on the regulation by IFNγ and TNFα, remain to be elucidated in future studies.

The results of this study demonstrate a significant association between GSRS and TEX levels in KIRC patients, which aligns with the established role of GCs in promoting TEX. GCs drive TEX primarily through sustained GR activation, initiating transcriptional programs that impair T cell function and induce hyporesponsiveness. Key mechanisms include upregulation of inhibitory receptors such as PD1, TIM3, and LAG3, which deliver negative signals that suppress T cell activation. Additionally, GCs repress effector cytokines including IL2, IFNγ, and TNFα, reducing cytotoxic activity. Epigenetic modifications, such as altered DNA methylation and histone acetylation at exhaustion-related gene loci, further stabilize the dysfunctional T cell phenotype. Transcription factors like BLIMP1 and TOX contribute to this process by promoting exhaustion-associated transcriptional networks. Together, enhanced inhibitory signaling, loss of effector function, and epigenetic reprogramming lead to diminished proliferative capacity and functional exhaustion in T cells [59].

Age and gender significantly influence treatment efficacy in KIRC, and their relevance to our findings merits further discussion. Thompson et al. reported that although younger patients (<40 years) more frequently presented with symptomatic tumors and chromophobe histology, no significant difference in cancer-specific survival was observed across age groups. Notably, older patients were more likely to undergo radical nephrectomy [60]. In contrast, our multivariate Cox analysis did not identify age as an independent prognostic factor, nor did we find significant correlation between GSRS and age. This discrepancy may reflect differences in cohort characteristics, evolving treatment modalities, or molecular specificity of the GSRS signature. Furthermore, sex-based disparities in immunotherapy response have been increasingly recognized. Incorvaia et al. observed that, despite generally stronger immune responses in females, those with clear cell histology, sarcomatoid differentiation, or under 50 years of age had shorter OS on immune-based combinations compared to males, with female gender being an independent negative prognostic factor in the sarcomatoid subgroup [61]. Although GSRS was not associated with gender in our study, we identified a positive correlation between serum GC levels and age. These distinctions highlight the complex interplay between sex hormones, immune response, and GC signaling in KIRC, suggesting that age and gender may modulate the TME and treatment efficacy through mechanisms not yet fully elucidated. Larger prospective studies are needed to clarify these relationships and assess whether integrating these factors could enhance prognostic stratification.

This study has several limitations that warrant consideration. First, while the efficacy of GSRS has been validated across multiple public datasets and local clinical samples, the retrospective design and relatively limited sample size of the local cohort may introduce potential biases and limit the generalizability of the findings. Future prospective studies with larger, diverse populations are needed to further validate these results. Second, the clinical implementation of GSRS faces key barriers, including the absence of standardized protocols and cost-effectiveness concerns, particularly in resource-limited settings. Future efforts should prioritize developing scalable and economically viable strategies to support its integration into routine practice. Third, a systematic head-to-head comparison of GSRS’s predictive performance against well-established biomarkers, such as IMDC score and PD-L1 expression, was not conducted. Future studies should explore explore the value of GSRS as a component of a composite predictive model, integrating it with factors like IMDC and PD-L1 to guide the application of GCs in combination with targeted therapies or ICIs. Fourth, although our animal experiments demonstrated that CORT supplementation can counteract the TEX caused by NFKB2 knockdown, it remains necessary to employ comprehensive approaches such as single-cell sequencing to assess changes in the infiltration and functional states of other immune cells. Last, although our findings suggest a potential regulatory relationship between GCs and key genes including ACADM, ANGPTL4, and NFKB2, the precise molecular mechanisms underlying these interactions remain unclear. Further mechanistic studies, particularly in the context of KIRC, are essential to elucidate these pathways and their functional implications.

## Conclusion

Patients with KIRC demonstrated elevated serum GC levels, which were significantly correlated with the degree of malignancy in KIRC. Furthermore, we developed a GC signaling-related gene signature to assess prognosis and predict immunotherapeutic response in KIRC, offering a valuable tool for guiding personalized treatment strategies.

## Supporting information

S1 Fig Unsupervised clustering of TCGA-KIRC subjects based on glucocorticoid signaling-related genes. (**A**) Non-negative matrix factorization (NMF) clustering was performed to determine the optimal number of clusters using cophenetic analysis. (**B**) Consensus matrix illustrating the clustering results. (**C**) Kaplan-Meier survival analysis revealed that C2 subjects had significantly worse overall survival (OS) compared to C1 cases. (**D**) C2 samples demonstrated a higher activation level of glucocorticoid signaling. (**E**) Differentially expressed genes (DEGs) between C1 and C2 clusters. (**F**) Functional enrichment analysis of DEGs between C1 and C2 samples, conducted using the Metascape database (https://metascape.org/gp/index.html#/main/step1).
(TIF)

S2 Fig Quantification analysis of the IHC staining from the HPA. (TIF)

S3 Fig Association between gender and the infiltration levels of NFKB2^+^ (A), ACADM^+^ (B), and ANGPTL4^+^ (C) cells in the local tissue samples. (TIF)

S4 Fig Pan-cancer analyses of ACADM expression and its prognostic significance in the TCGA database. (**A**) Differential expression of ACADM between paracarcinoma and tumor tissues across various cancer types. (**B**) Predictive ability of ACADM expression for OS in pan-cancer analyses.
(TIF)

S5 Fig Pan-cancer analyses of ANGPTL4 expression and its prognostic significance in the TCGA database. (**A**) Differential expression of ANGPTL4 between paracarcinoma and tumor tissues across various cancer types. (**B**) Predictive ability of ANGPTL4 expression for OS in pan-cancer analyses.
(TIF)

S6 Fig Pan-cancer analyses of NFKB2 expression and its prognostic significance in the TCGA database. (**A**) Differential expression of NFKB2 between paracarcinoma and tumor tissues across various cancer types. (**B**) Predictive ability of NFKB2 expression for OS in pan-cancer analyses.
(TIF)

S7 Fig Meta-analyses were performed to aggregate the HRs for continuous (A) and binary (B) GSRS after excluding the GSE167573 cohort. (TIF)

S8 Fig Distribution (A) and association (B) between the identified clusters and the glucocorticoid signaling-related score (GSRS) stratification in the TCGA-KIRC cohort. (TIF)

S9 Fig Enhanced GC signaling response in high-GSRS KIRC samples. (TIF)

S10 Fig The predictive efficacy of GSRS in combination with GR and/or HSD11B1 for the prognosis of KIRC patients. (**A**) Kaplan-Meier survival analyses indicated the association of GR or HSD11B1 expressions with OS in the TCGA-KIRC cohort. The optimal cut-off values were detected by the X-tile. (**B**) The levels of GR or HSD11B1 in the normal kidney and KIRC tissues, which were obtained from the HPA. (**C**) Time-dependent ROC analyses indicated the predictive ability of GSRS in combination with GR and/or HSD11B1 for 1-, 2-, 3–4-, and 5-year OS.
(TIF)

S11 Fig Pan-cancer analyses of GSRS. (**A**) Tumor samples showed significantly higher levels of GSRS in 16 out of 33 cancer types compared to adjacent paracarcinoma tissues, indicating a potential role of glucocorticoid signaling in tumorigenesis. (**B**) The predictive ability of GSRS for OS across a pan-cancer level, demonstrating its potential as a prognostic biomarker. (**C-D**) Higher GSRS levels were associated with unfavorable OS in kidney renal papillary cell carcinoma (KIRP) (**C**) and acute myeloid leukemia (LAML) (**D**), suggesting a negative prognostic impact in these cancers. (**E-F**) Conversely, higher GSRS levels were linked to favorable OS in lower grade glioma (LGG) (**E**) and sarcoma (SARC) (**F**), indicating a potential protective or beneficial role in these cancer types.
(TIF)

S12 Fig Meta-analysis revealed a positive association between GSRS and TNF-α signaling across 8 KIRC cohorts. (TIF)

S13 Fig Association between GSRS and CD8^+^ T cell infiltration levels in the TCGA-KIRC cohort, and the infiltration proportion of CD8^+^ T cells was evaluated using multiple deconvolution algorithms, including TIMER (A), CIBERSORT (B), QUANTISEQ (C), MCPCOUNTER (D), and XCELL (E). (TIF)

S14 Fig Spatial transcriptomics analysis indicated the association of GSRS with CD8^+^ T cell infiltration and TEX in KIRC samples. (TIF)

S15 Fig Comparison of the predictive performance of GSRS, tumor mutational burden (TMB), and microsatellite instability (MSI) in forecasting immunotherapeutic response, as assessed by the tumor immune dysfunction and exclusion (TIDE) algorithm, within the TCGA-KIRC cohort. (TIF)

S16 Fig Comparison of the predictive performance of GSRS and the expression levels of immune checkpoint genes in relation to progression-free survival (PFS) in the subjects treated with Avelumab in combination with Axitinib within the JAVELIN Renal 101 cohort. (TIF)

S17 Fig The top 20 candidate drugs demonstrating the most significant sensitivity differences between high- and low-GSRS subjects. These include ULK1_4989 (A), Mirin (B), XAV939 (C), Vinorelbine (D), Epirubicin (E), GNE-317 (F), Topotecan (G), Irinotecan (H), SB505124 (I), Camptothecin (J), Elephantin (K), AZD7762 (L), MG-132 (M), Palbociclib (N), 5-Fluorouracil (O), Vinblastine (P), Rapamycin (Q), Teniposide (R), Pictilisib (S), and PRIMA-1MET (T).
(TIF)

S18 Fig The predicted IC50 values of Axitinib in the low- and high-GSRS cases. (TIF)

S19 Fig Spatial transcriptomics analysis of NFKB2, HSD11B1, and GR in KIRC tissues. (TIF)

S20 Fig Real-time quantitative PCR (RT-qPCR) analysis was performed to validate the knockdown efficiency of NFKB2 in Renca cells. (**A**) Melting curve plots for detecting NFKB2 and GAPDH. (**B**) The relative expressions of NFKB2 in diverse Renca cells.
(TIF)

S21 Fig Immunohistochemical (IHC) staining was conducted to assess NFKB2 expression levels in tumor tissues obtained from mice across different experimental groups. (TIF)

S22 Fig The AUCs of the GSRS and the established models in predicting the 1-, 2-, 3-, 4-, and 5-year OS in the TCGA-KIRC cohort. (TIF)

S1 Table The baseline clinicopathological features of the public and local KIRC cohorts. (XLSX)

S2 Table 655 glucocorticoid signaling-related genes collected from the MSigDB. (XLSX)

S3 Table The primer sequence used in this study. (XLSX)

S4 Table The glucocorticoid signaling-related genes showing differentially expression between paracarcinoma and KIRC tissues. (XLSX)

S5 Table The strength of gene interactions within this PPI network. (XLSX)

S6 Table The functional enrichment of the glucocorticoid signaling-related genes showing differential expression through the Metascape. (XLSX)

S7 Table The differentially-expressed genes between C1 and C2 cases. (XLSX)

S8 Table The functional enrichment of the differentially-expressed genes between C1 and C2 cases through the Metascape. (XLSX)

S9 Table The coefficients of the features in the LASSO regression model. (XLSX)

S10 Table Pan-cancer analyses of the prognosis value of ACADM. (XLSX)

S11 Table Pan-cancer analyses of the prognosis value of ANGPTL4. (XLSX)

S12 Table Pan-cancer analyses of the prognosis value of NFKB2. (XLSX)

S13 Table Pan-cancer analyses of the prognosis value of GSRS. (XLSX)

S14 Table The correlation between GSRS and the activities of immune-related pathways in KIRC cohorts. (TIF)

S15 Table Evaluation of the association between GSRS and CD8^+^ T cell infiltration using multiple algorithms based on the TCGA-KIRC cohort. (XLSX)

S16 Table Pan-cancer analyses of the association between GSRS and CD8^+^ T cell infiltration using the CIBERSORT-ABS algorithm. (XLSX)

S17 Table The association between GSRS and the expressions of TEX-related genes in KIRC cohorts. (XLSX)

S18 Table The drug sensitivity predicted by oncopredict in the TCGA-KIRC cohort. (XLSX)

S19 Table The difference of drug sensitivity between high and low-GSRS subjects in the TCGA-KIRC cohort. (XLSX)

S20 Table The tumor volume (mm^3^) of the mice in each group. (XLSX)

S21 Table The 44 gene signatures collected from the published literature. (XLSX)

S1 File Detailed materials and methods. (DOCX)
