# Supplementary figures and images for: Developing and experimentally validating a glucocorticoid signaling-related gene signature to evaluate the prognosis and immunotherapeutic response in kidney renal clear cell carcinoma
Source: PLoS One. 2025 Oct 13;20(10):e0334104. doi: 10.1371/journal.pone.0334104 (PMC12517536; doi:10.1371/journal.pone.0334104)

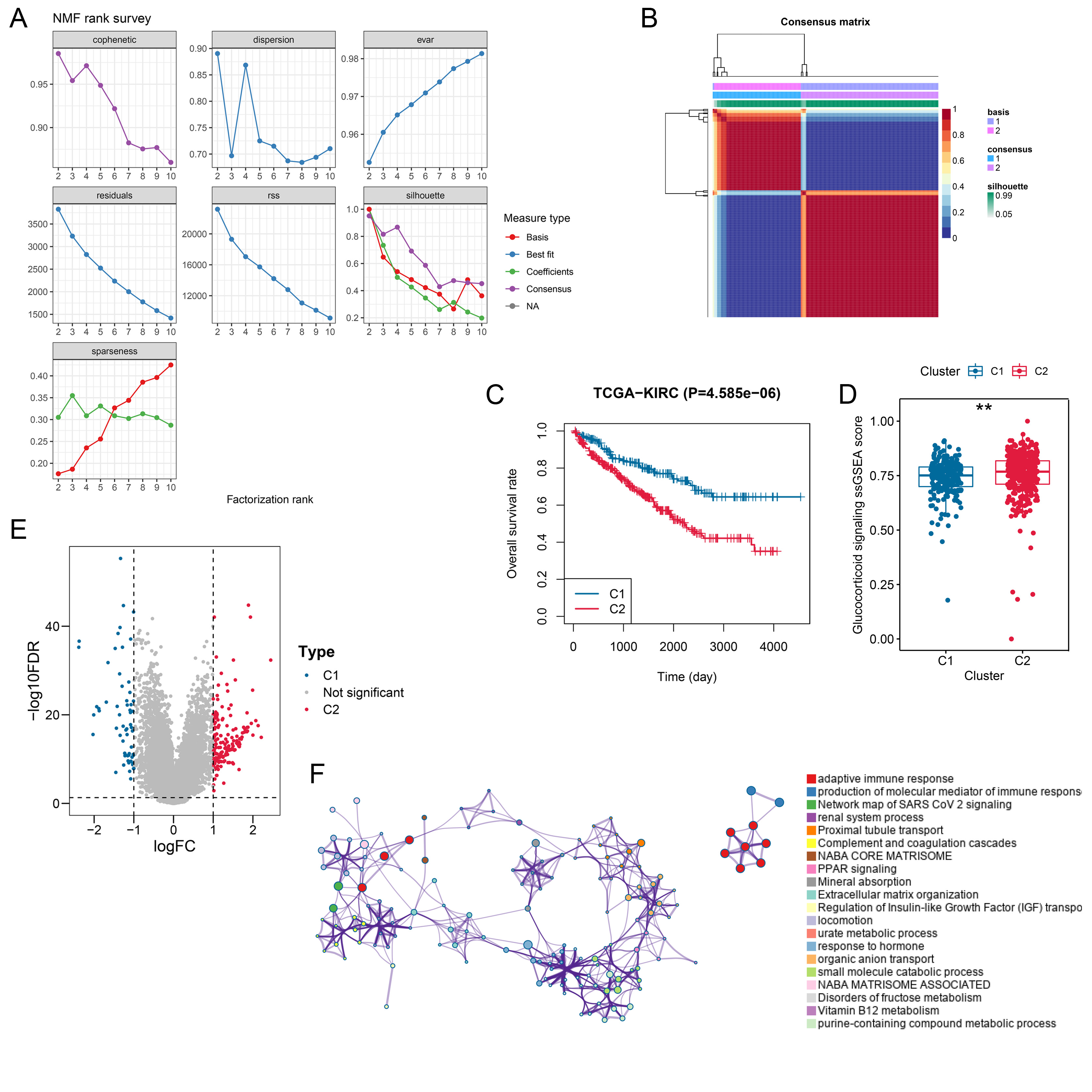

Supplement: S1 Fig — (A) Non-negative matrix factorization (NMF) clustering was performed to determine the optimal number of clusters using cophenetic analysis. (B) Consensus matrix illustrating the clustering results. (C) Kaplan-Meier survival analysis revealed that C2 subjects had significantly worse overall survival (OS) compared to C1 cases. (D) C2 samples demonstrated a higher activation level of glucocorticoid signaling. (E) Differentially expressed genes (DEGs) between C1 and C2 clusters. (F) Functional enrichment analysis of DEGs between C1 and C2 samples, conducted using the Metascape database (https://metascape.org/gp/index.html#/main/step1). (TIF) [file pone.0334104.s001.tif]

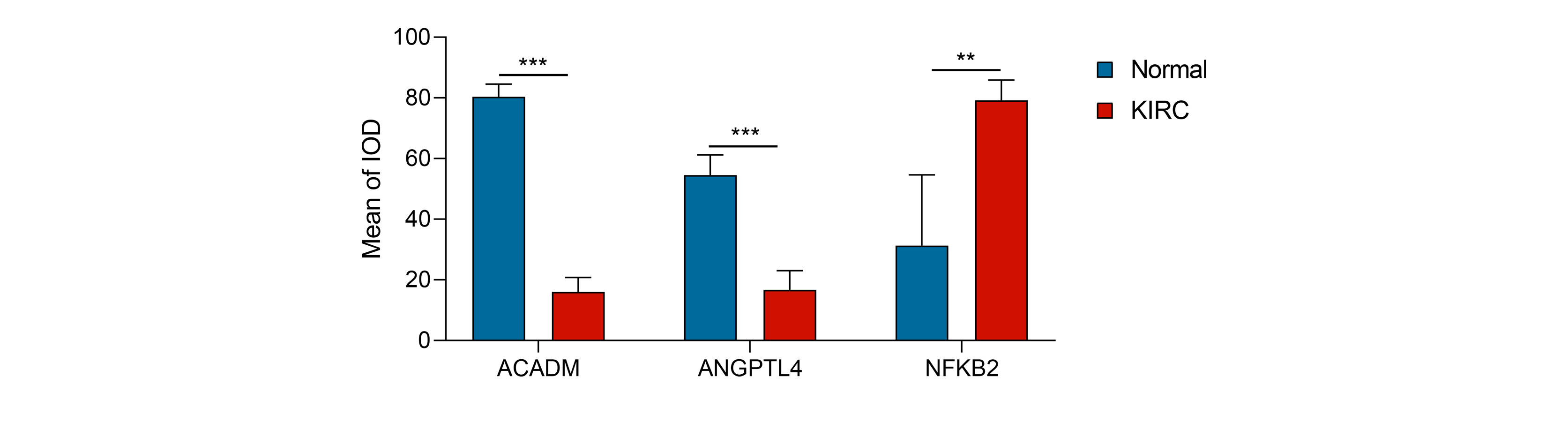

Supplement: S2 Fig — (TIF) [file pone.0334104.s002.tif]

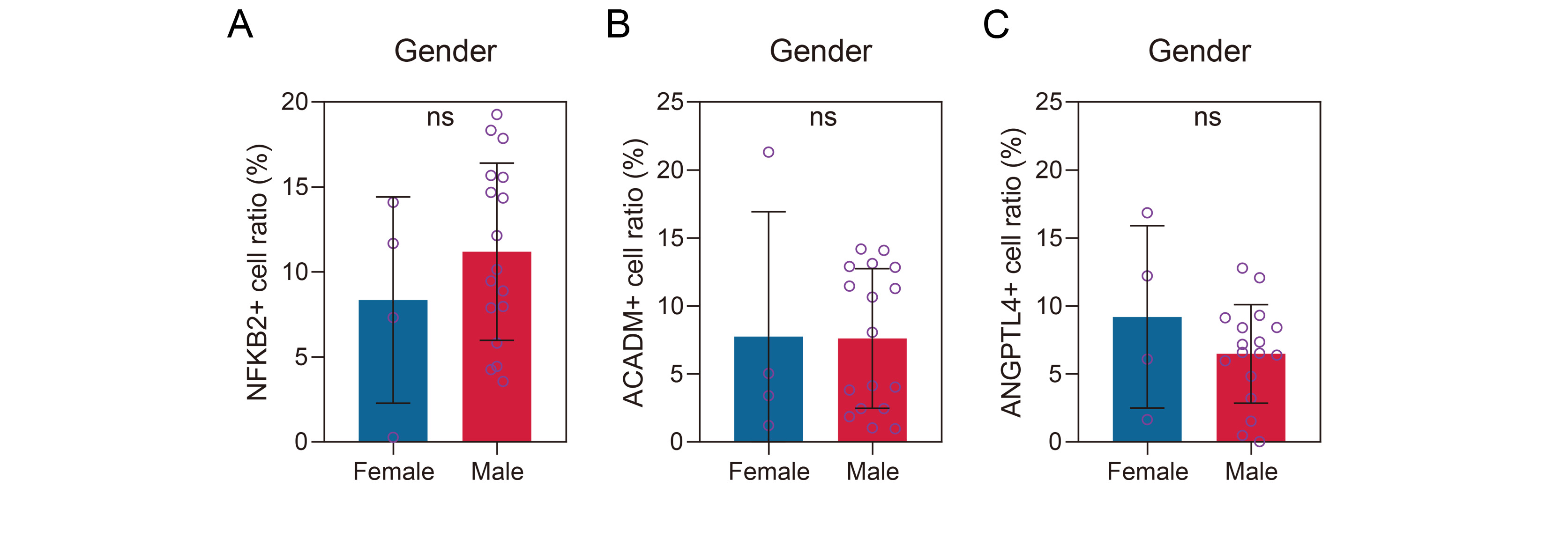

Supplement: S3 Fig — (TIF) [file pone.0334104.s003.tif]

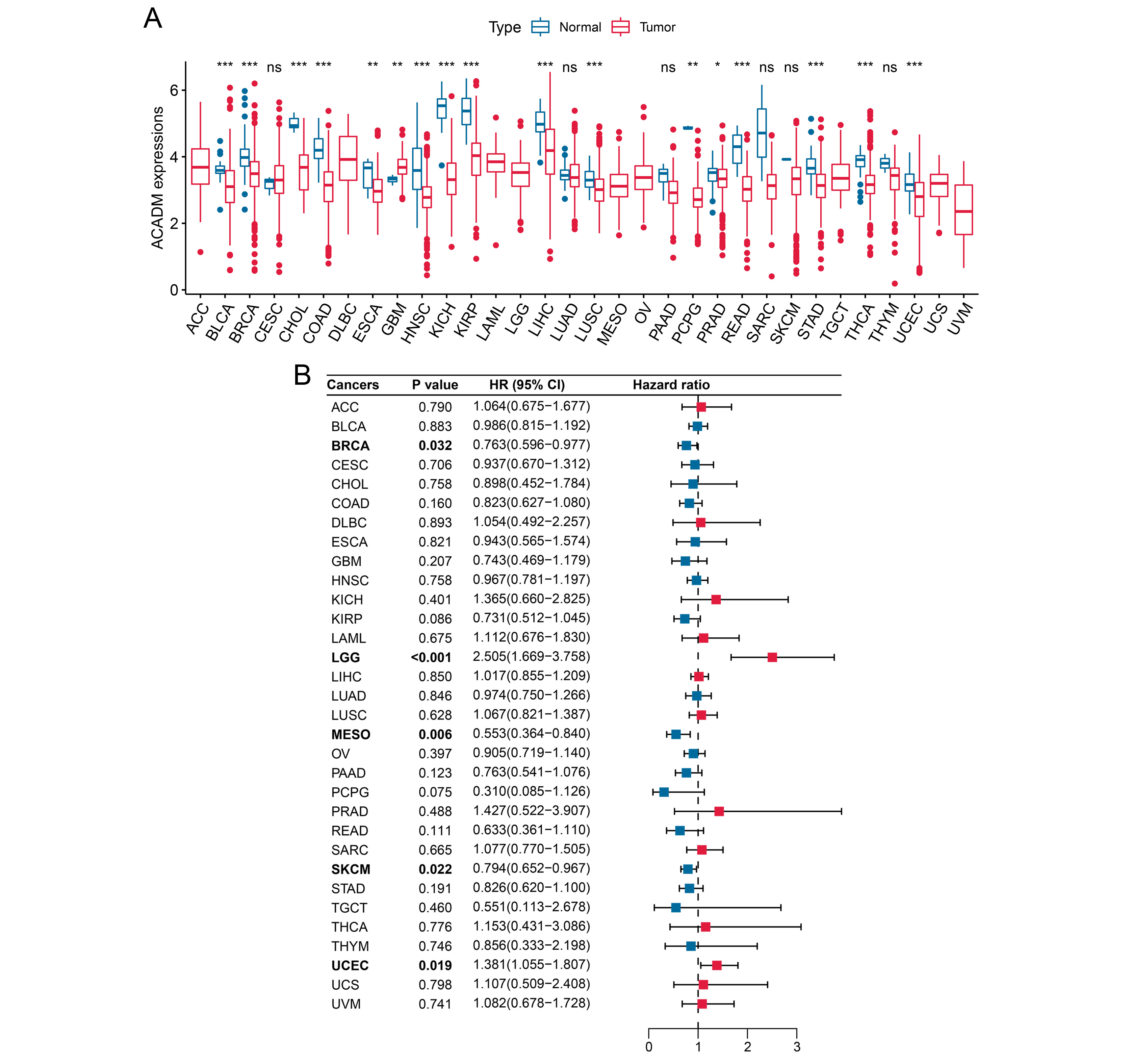

Supplement: S4 Fig — (A) Differential expression of ACADM between paracarcinoma and tumor tissues across various cancer types. (B) Predictive ability of ACADM expression for OS in pan-cancer analyses. (TIF) [file pone.0334104.s004.tif]

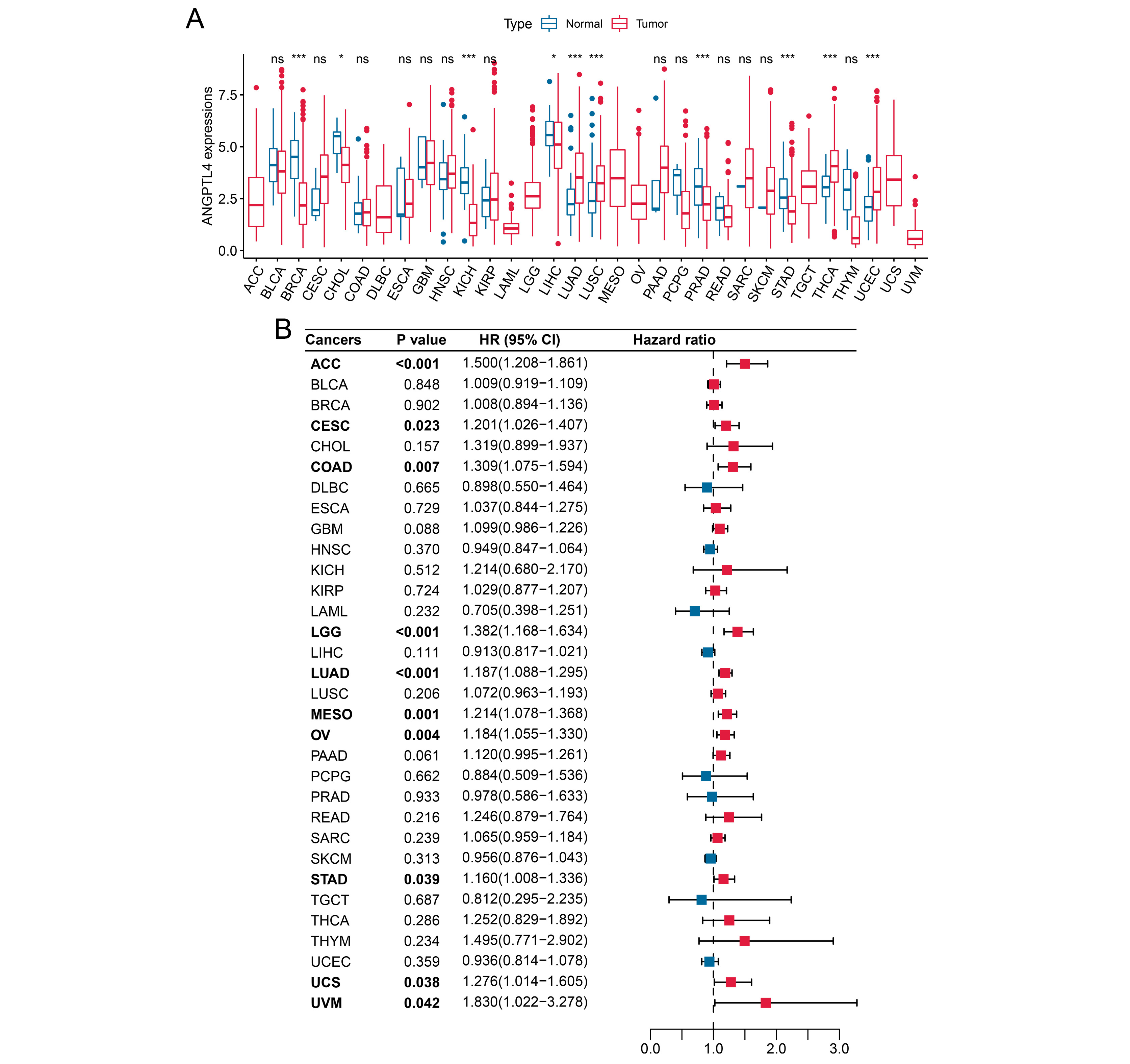

Supplement: S5 Fig — (A) Differential expression of ANGPTL4 between paracarcinoma and tumor tissues across various cancer types. (B) Predictive ability of ANGPTL4 expression for OS in pan-cancer analyses. (TIF) [file pone.0334104.s005.tif]

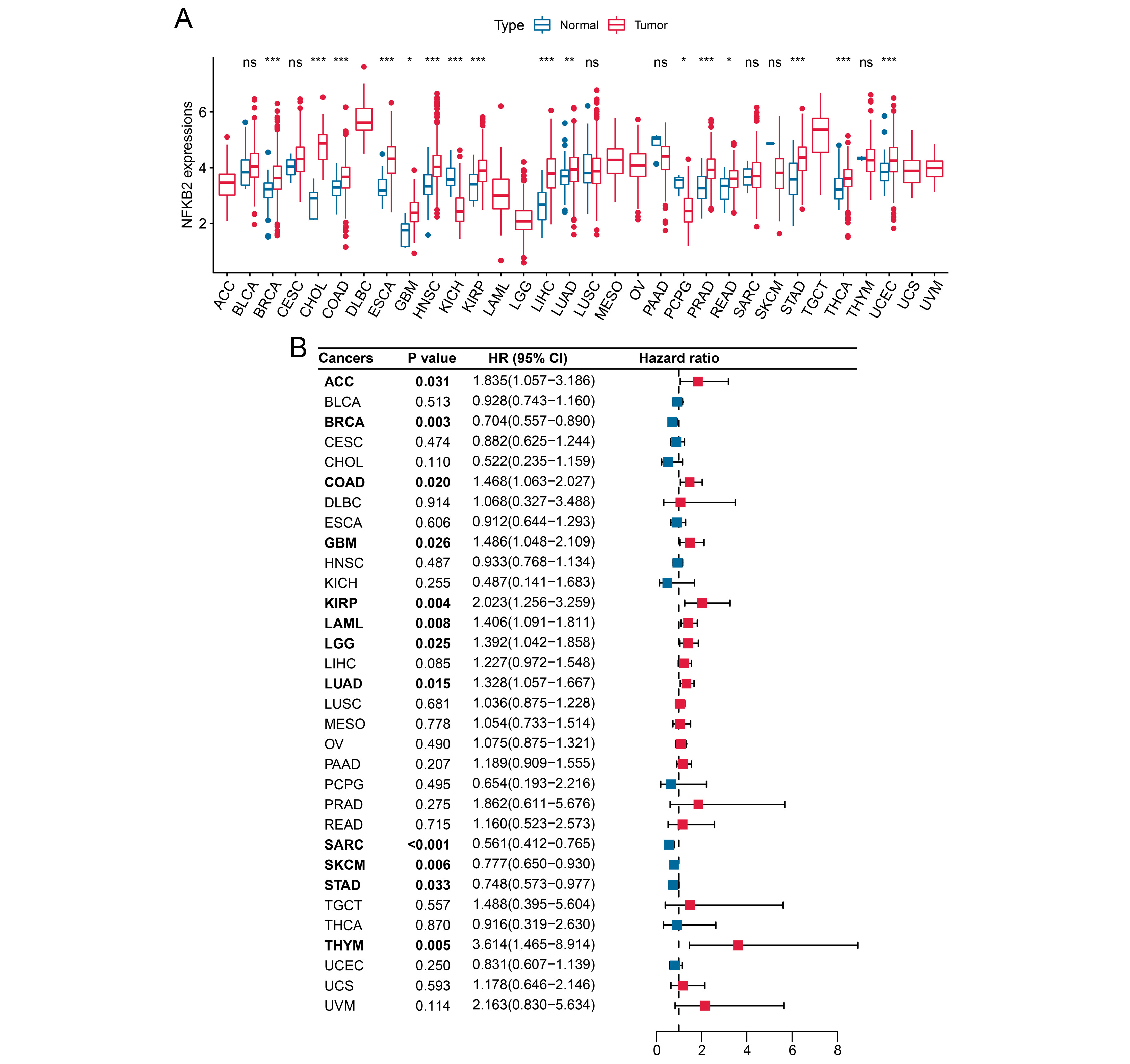

Supplement: S6 Fig — (A) Differential expression of NFKB2 between paracarcinoma and tumor tissues across various cancer types. (B) Predictive ability of NFKB2 expression for OS in pan-cancer analyses. (TIF) [file pone.0334104.s006.tif]

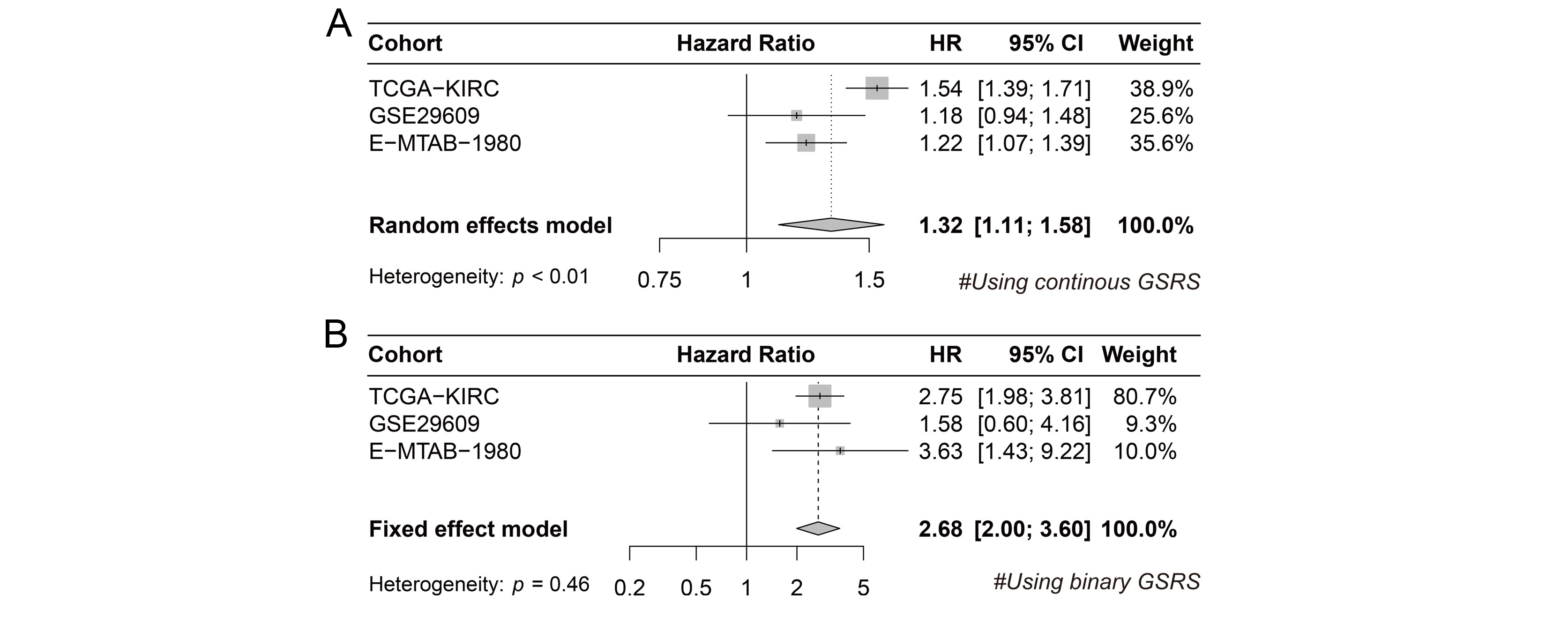

Supplement: S7 Fig — (TIF) [file pone.0334104.s007.tif]

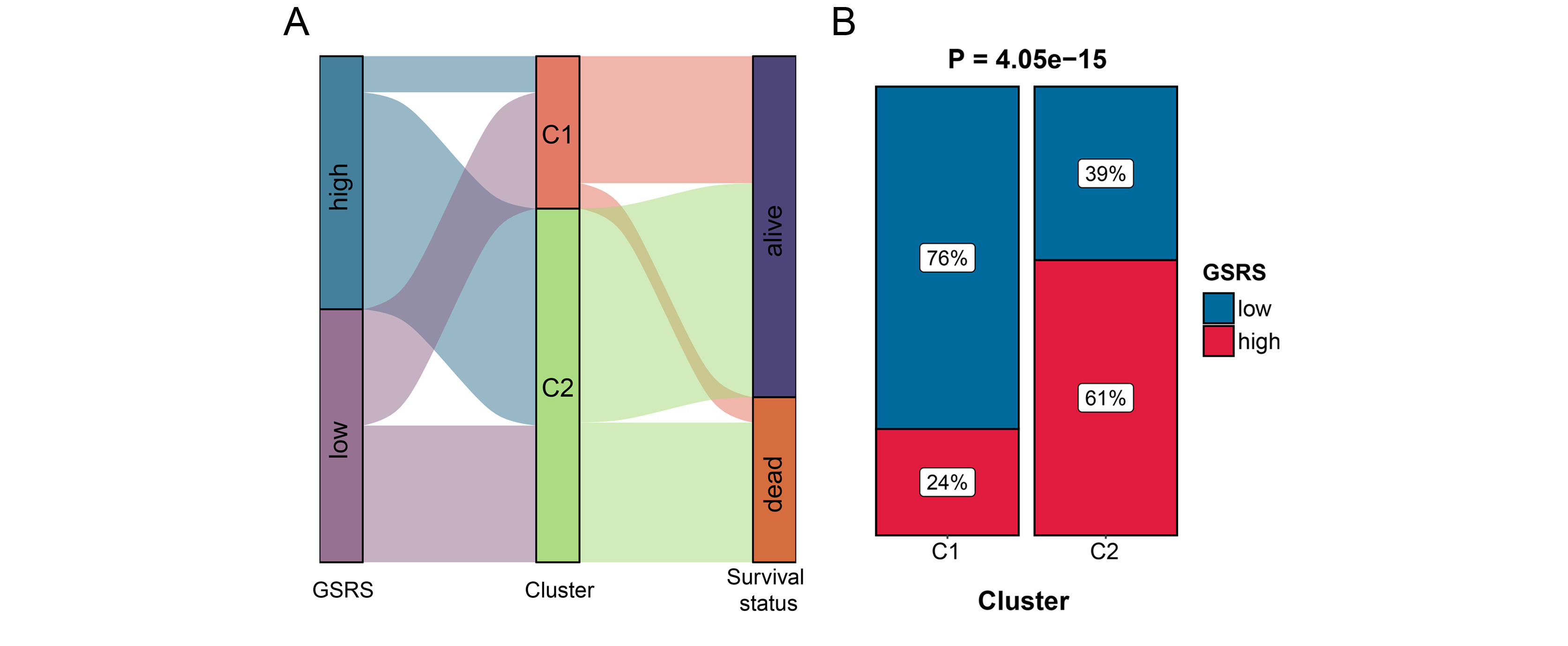

Supplement: S8 Fig — (TIF) [file pone.0334104.s008.tif]

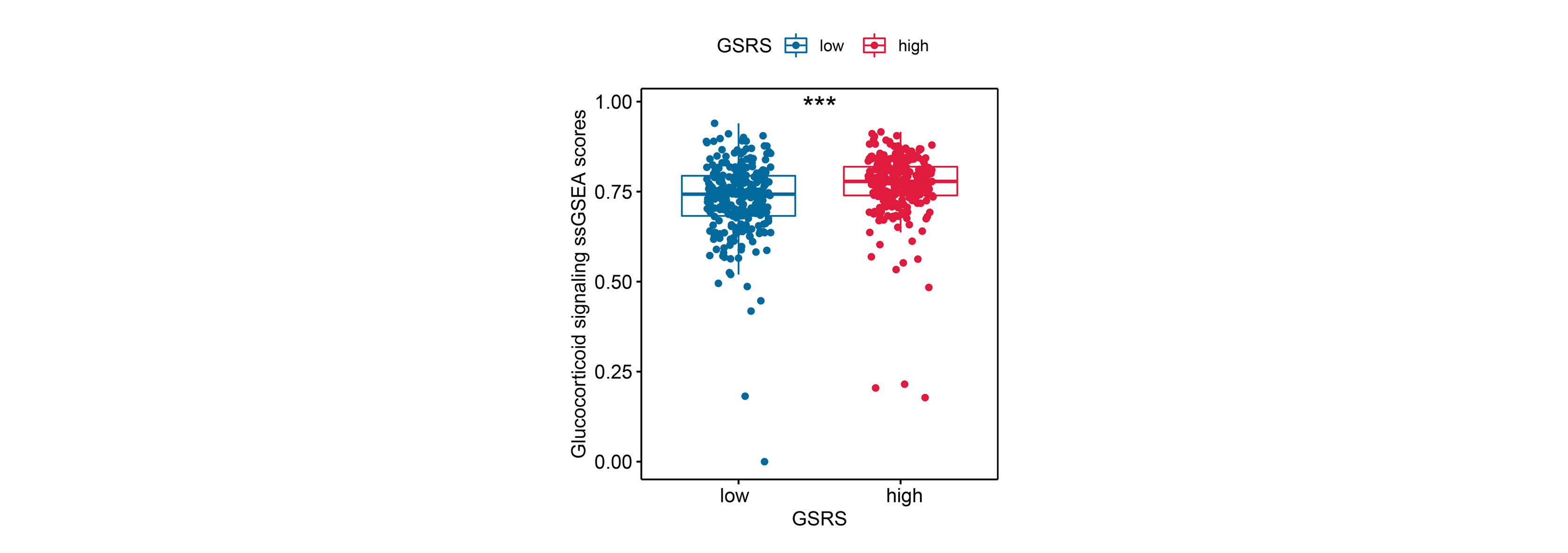

Supplement: S9 Fig — (TIF) [file pone.0334104.s009.tif]

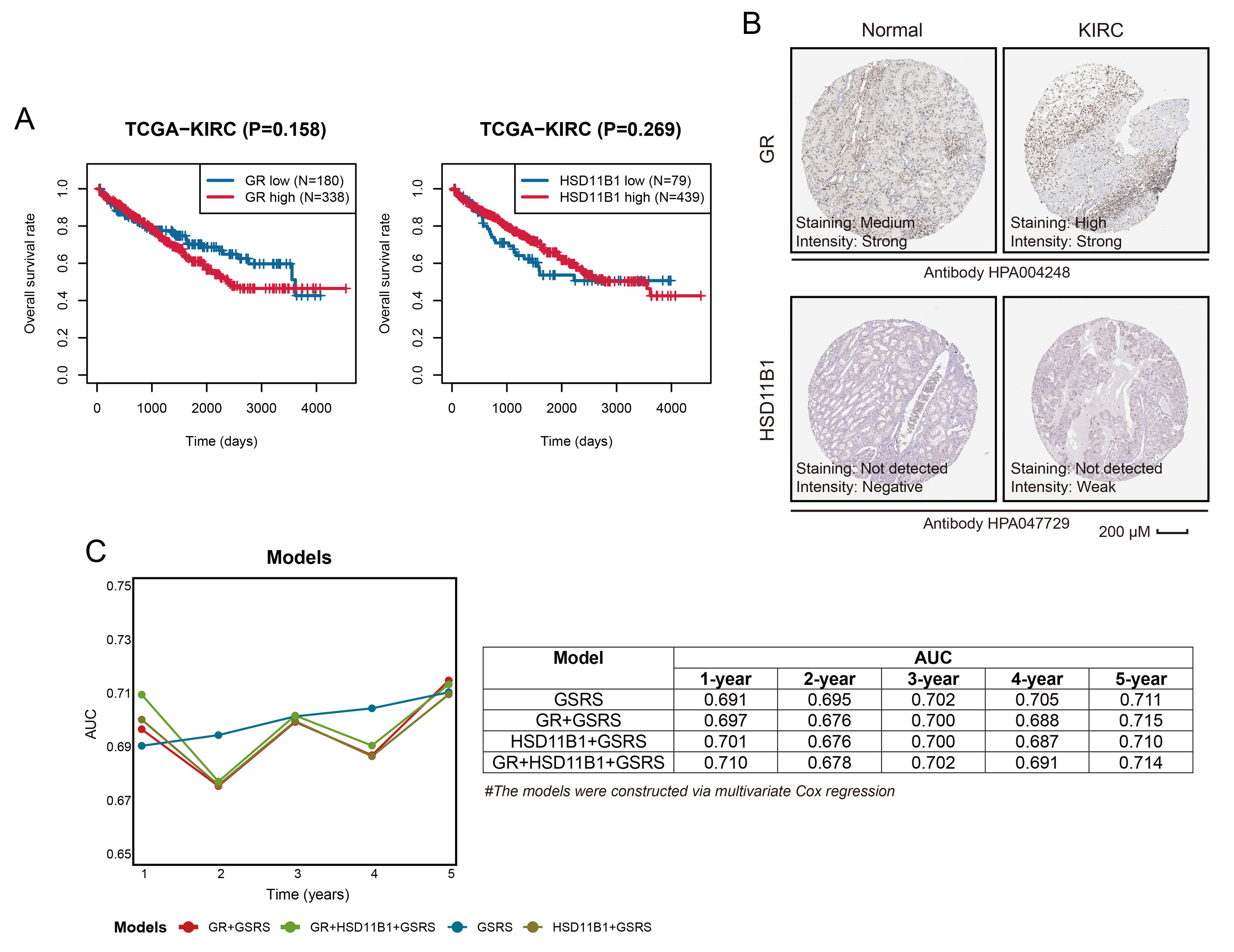

Supplement: S10 Fig — (A) Kaplan-Meier survival analyses indicated the association of GR or HSD11B1 expressions with OS in the TCGA-KIRC cohort. The optimal cut-off values were detected by the X-tile. (B) The levels of GR or HSD11B1 in the normal kidney and KIRC tissues, which were obtained from the HPA. (C) Time-dependent ROC analyses indicated the predictive ability of GSRS in combination with GR and/or HSD11B1 for 1-, 2-, 3–4-, and 5-year OS. (TIF) [file pone.0334104.s010.tif]

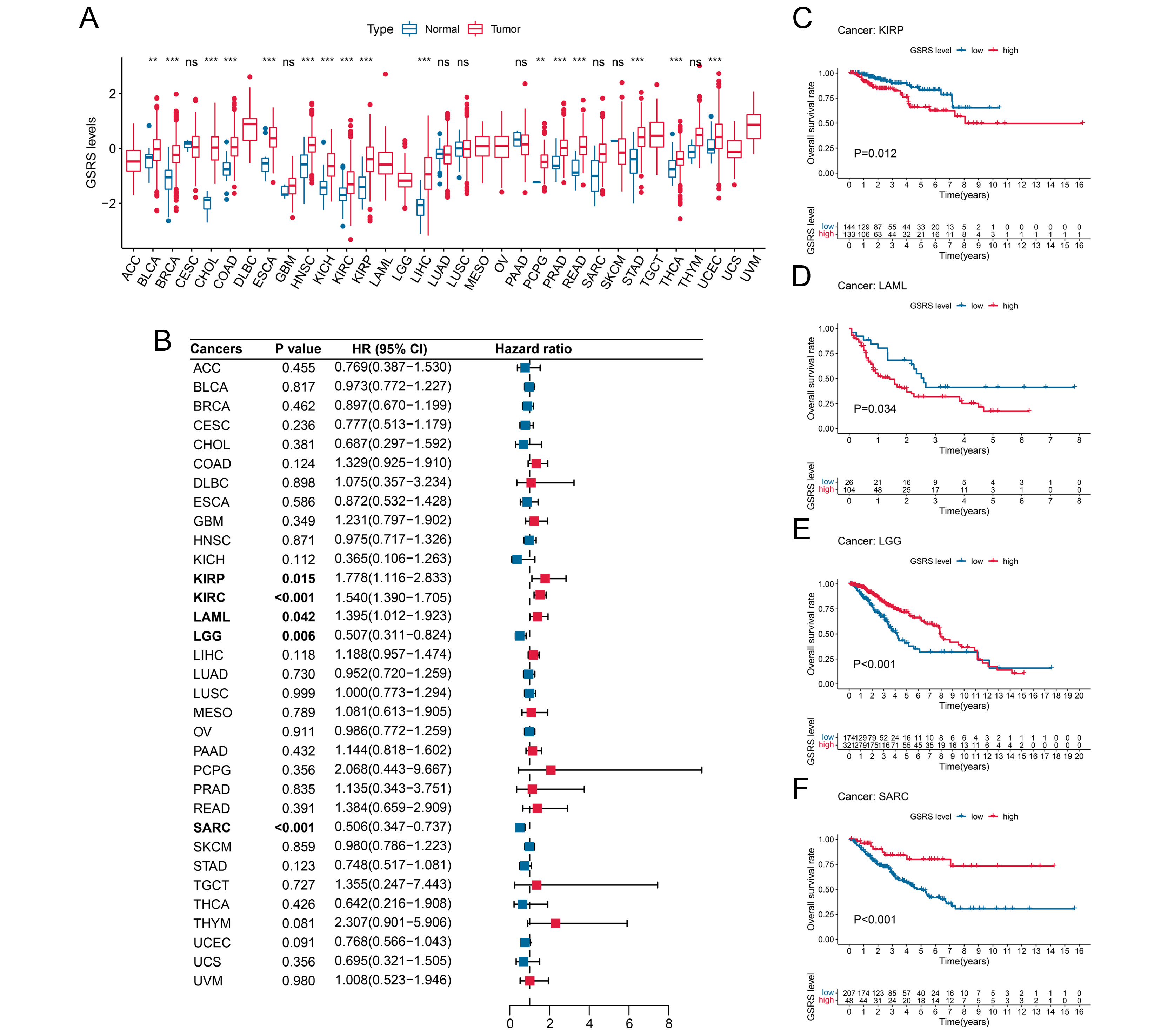

Supplement: S11 Fig — (A) Tumor samples showed significantly higher levels of GSRS in 16 out of 33 cancer types compared to adjacent paracarcinoma tissues, indicating a potential role of glucocorticoid signaling in tumorigenesis. (B) The predictive ability of GSRS for OS across a pan-cancer level, demonstrating its potential as a prognostic biomarker. (C-D) Higher GSRS levels were associated with unfavorable OS in kidney renal papillary cell carcinoma (KIRP) (C) and acute myeloid leukemia (LAML) (D), suggesting a negative prognostic impact in these cancers. (E-F) Conversely, higher GSRS levels were linked to favorable OS in lower grade glioma (LGG) (E) and sarcoma (SARC) (F), indicating a potential protective or beneficial role in these cancer types. (TIF) [file pone.0334104.s011.tif]

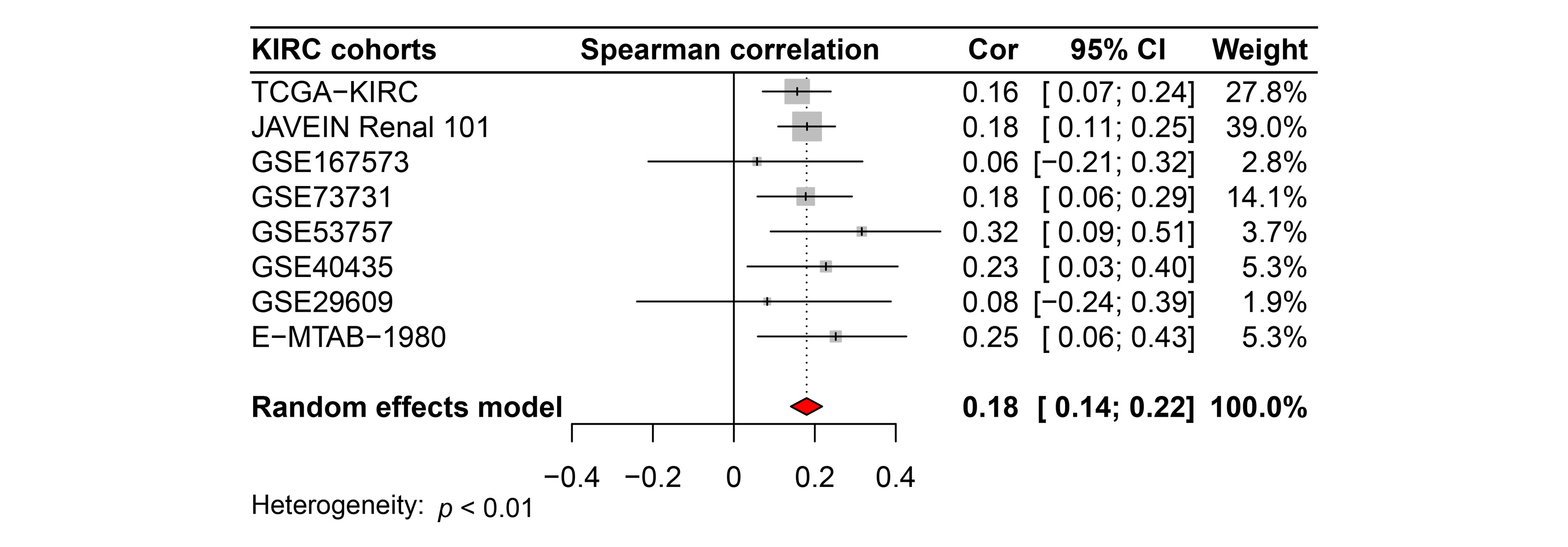

Supplement: S12 Fig — (TIF) [file pone.0334104.s012.tif]

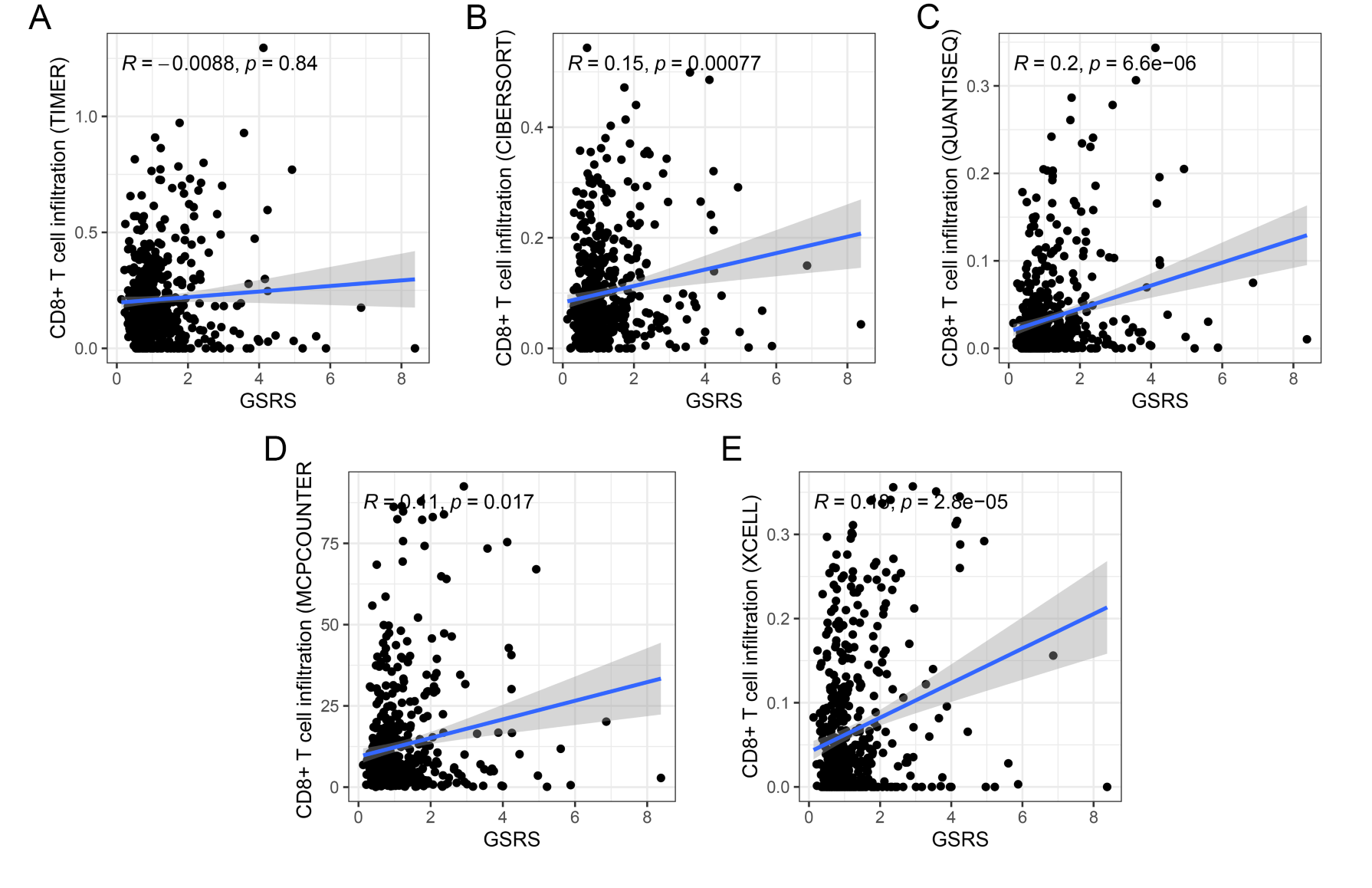

Supplement: S13 Fig — (TIF) [file pone.0334104.s013.tif]

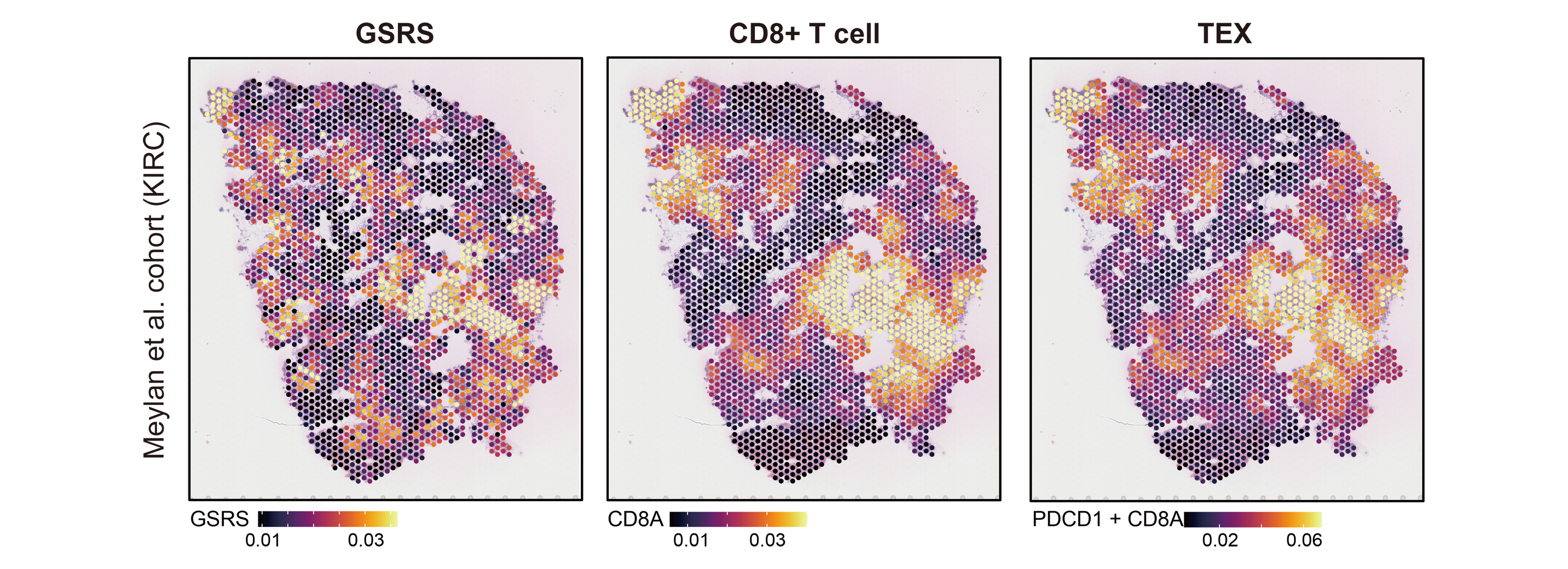

Supplement: S14 Fig — (TIF) [file pone.0334104.s014.tif]

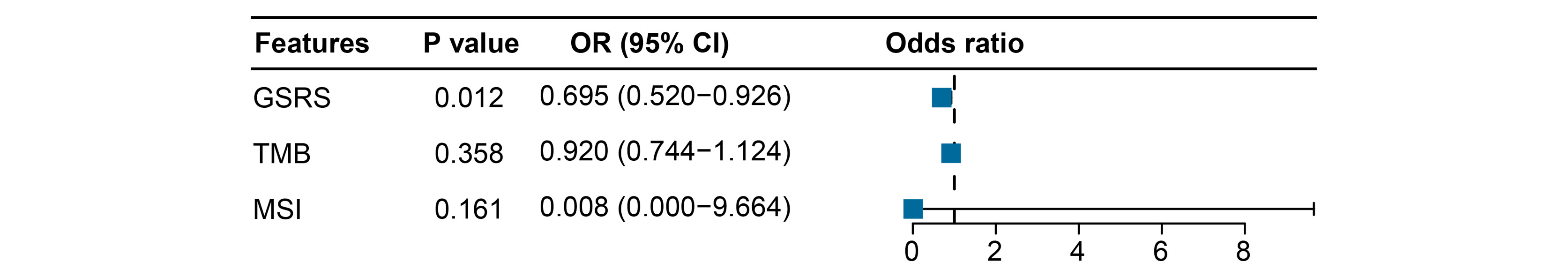

Supplement: S15 Fig — (TIF) [file pone.0334104.s015.tif]

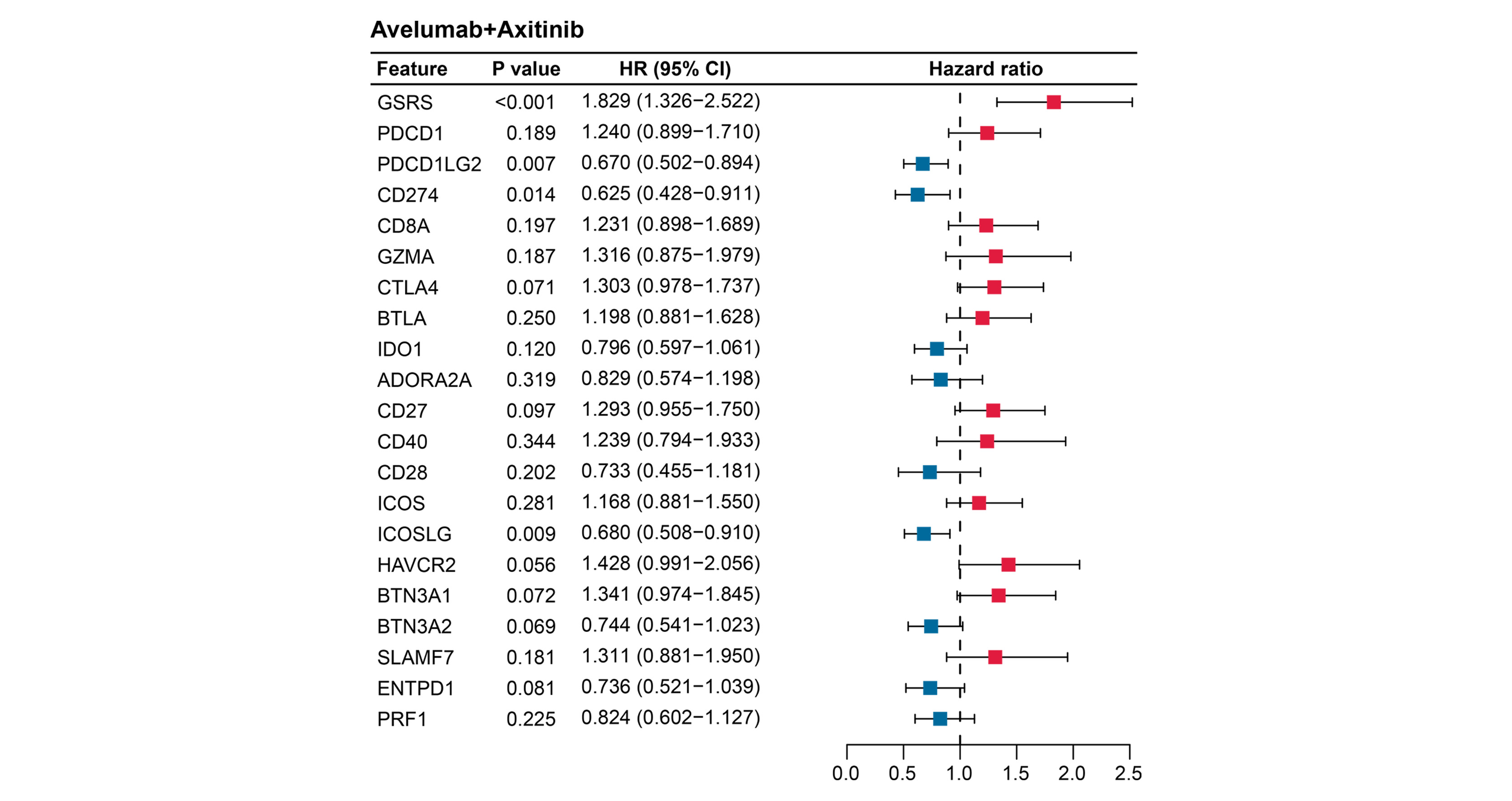

Supplement: S16 Fig — (TIF) [file pone.0334104.s016.tif]

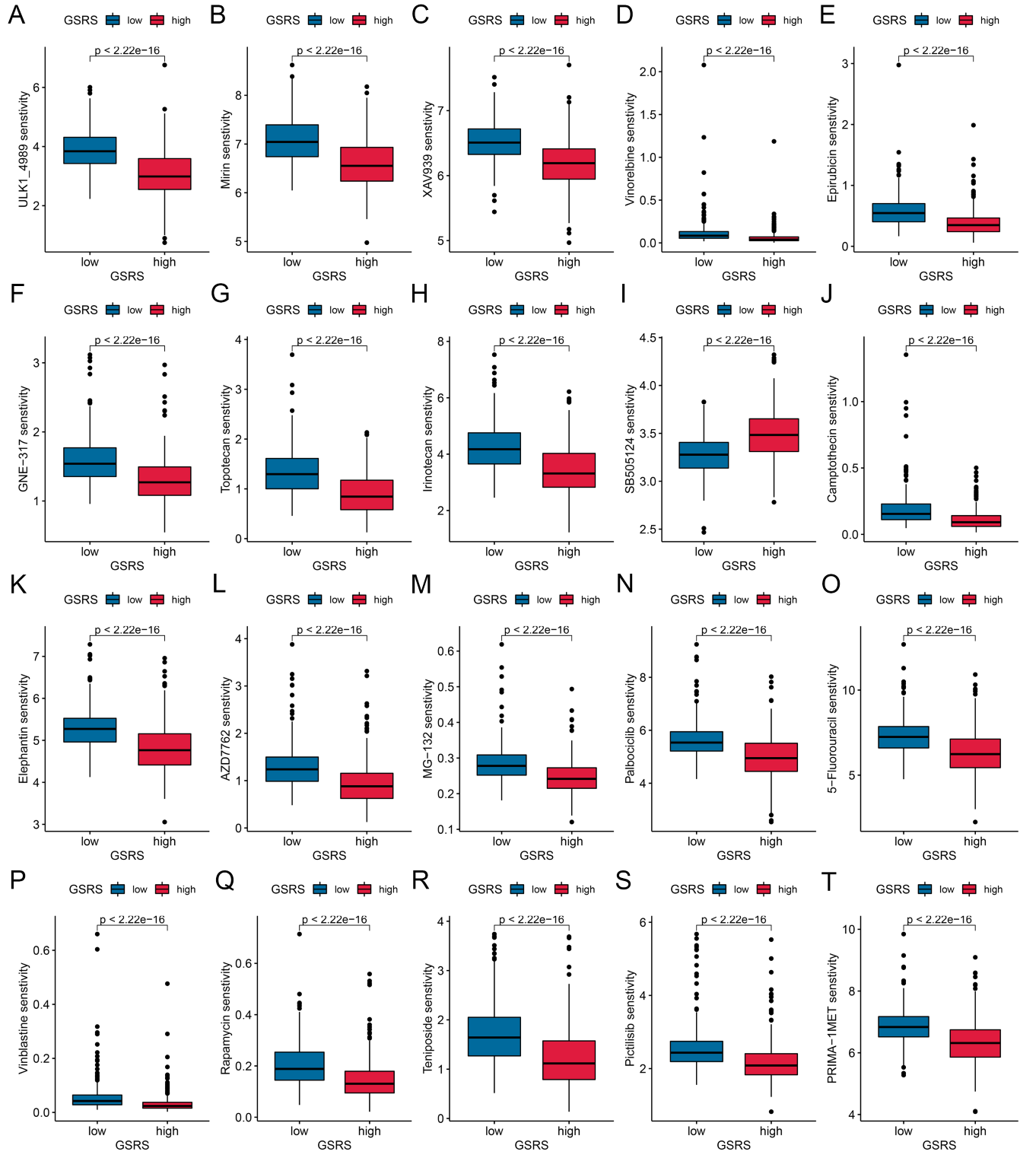

Supplement: S17 Fig — These include ULK1_4989 (A), Mirin (B), XAV939 (C), Vinorelbine (D), Epirubicin (E), GNE-317 (F), Topotecan (G), Irinotecan (H), SB505124 (I), Camptothecin (J), Elephantin (K), AZD7762 (L), MG-132 (M), Palbociclib (N), 5-Fluorouracil (O), Vinblastine (P), Rapamycin (Q), Teniposide (R), Pictilisib (S), and PRIMA-1MET (T). (TIF) [file pone.0334104.s017.tif]

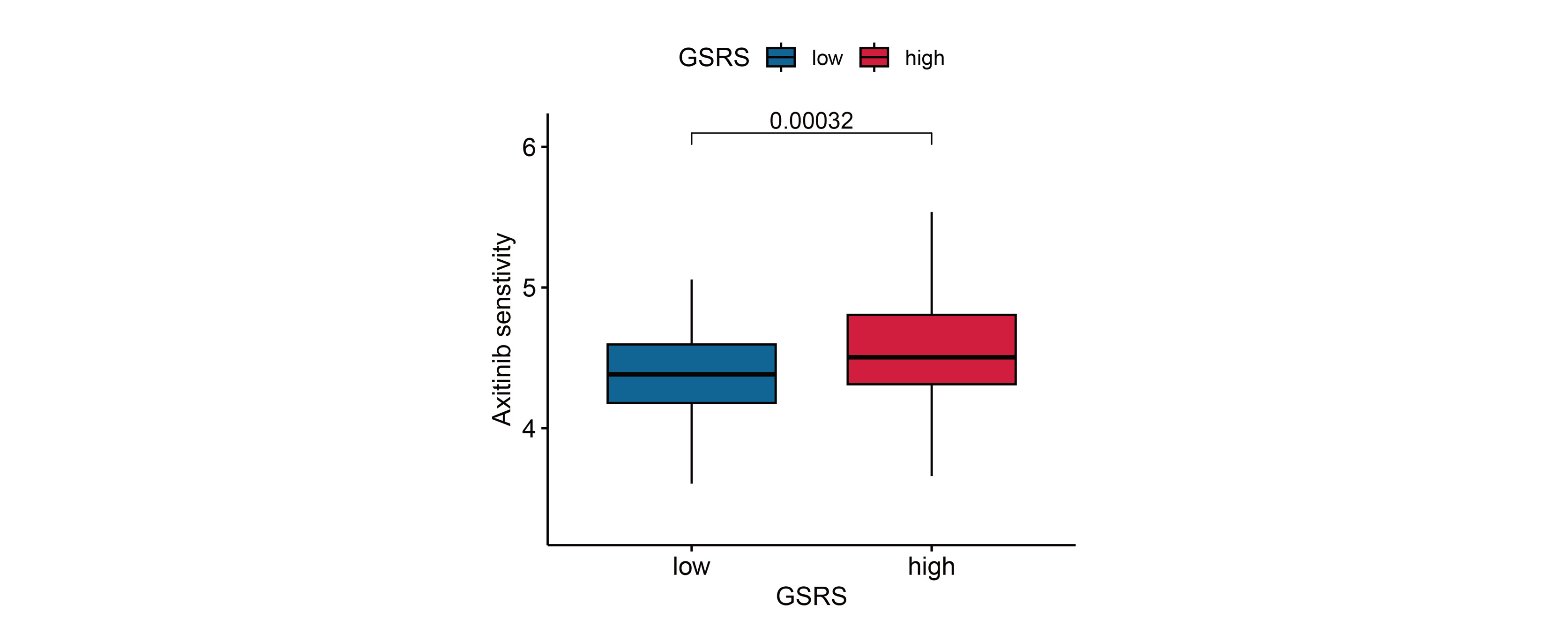

Supplement: S18 Fig — (TIF) [file pone.0334104.s018.tif]

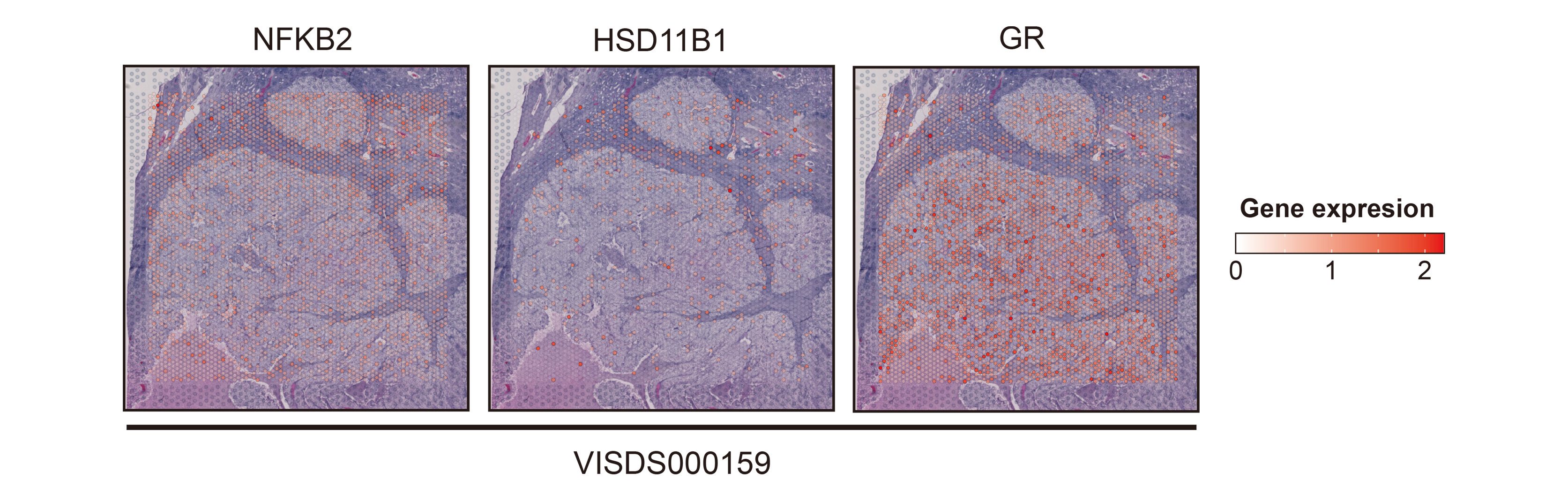

Supplement: S19 Fig — (TIF) [file pone.0334104.s019.tif]

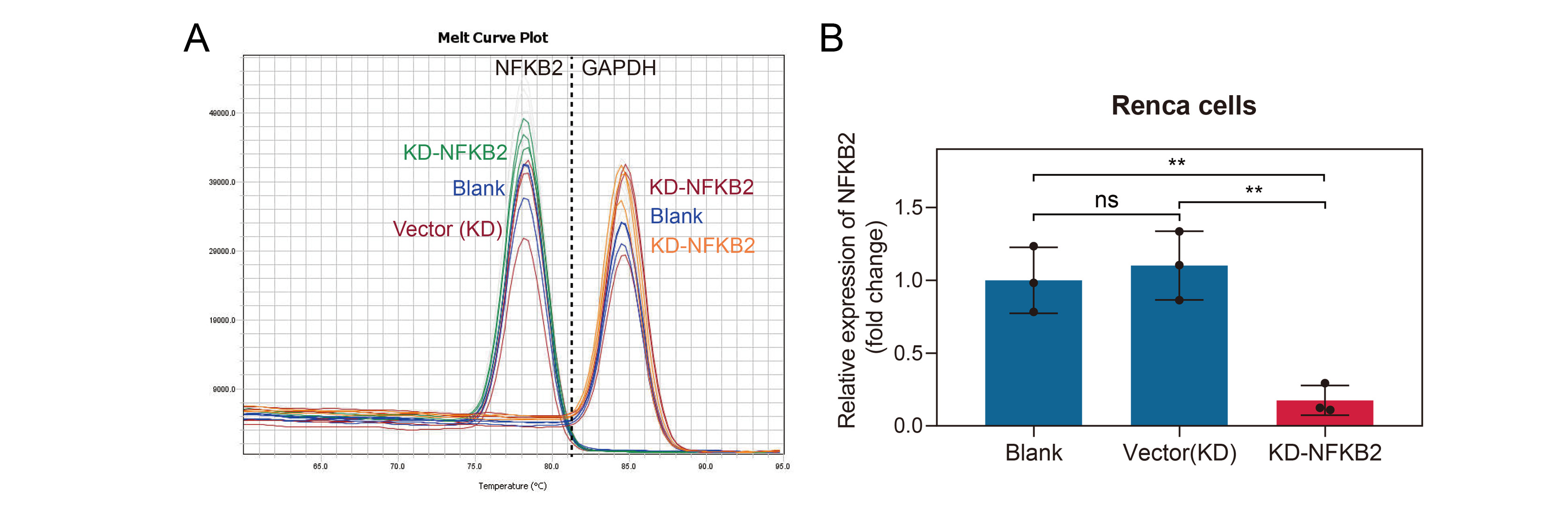

Supplement: S20 Fig — (A) Melting curve plots for detecting NFKB2 and GAPDH. (B) The relative expressions of NFKB2 in diverse Renca cells. (TIF) [file pone.0334104.s020.tif]

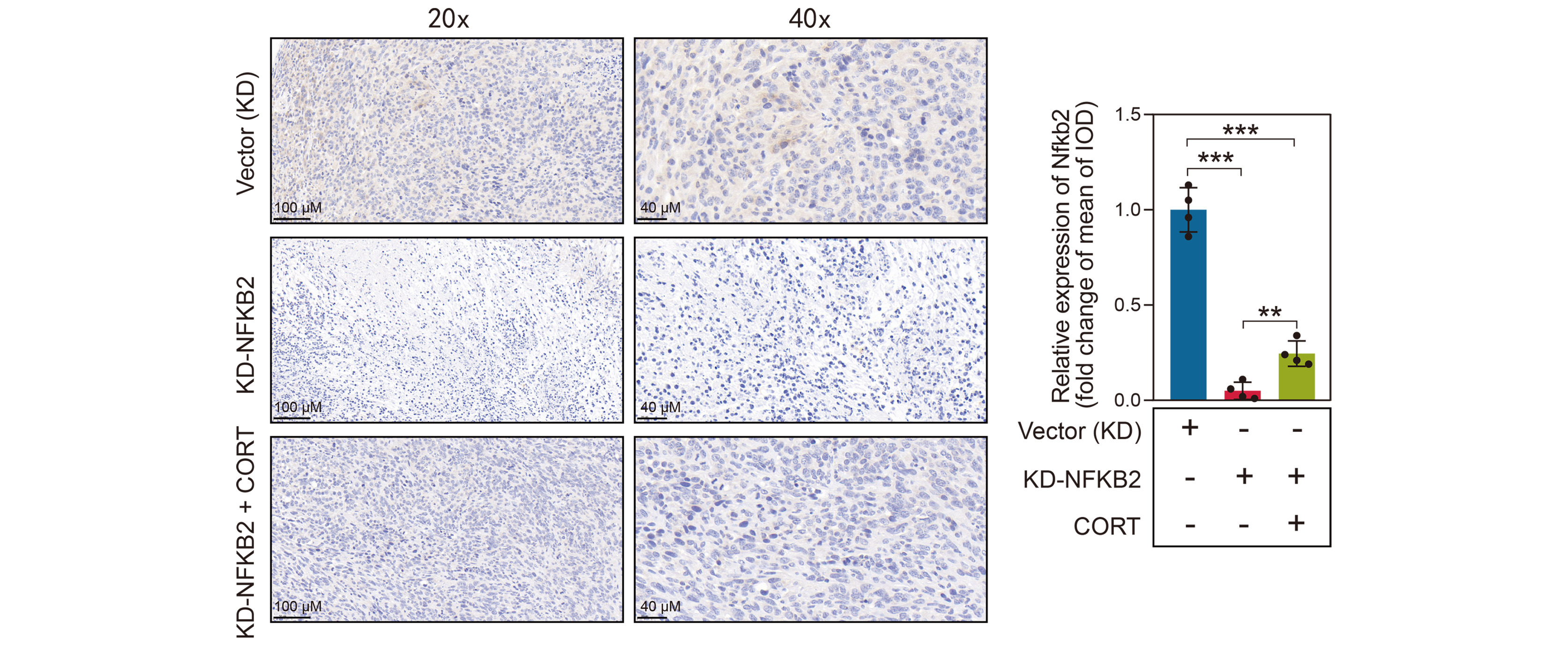

Supplement: S21 Fig — (TIF) [file pone.0334104.s021.tif]

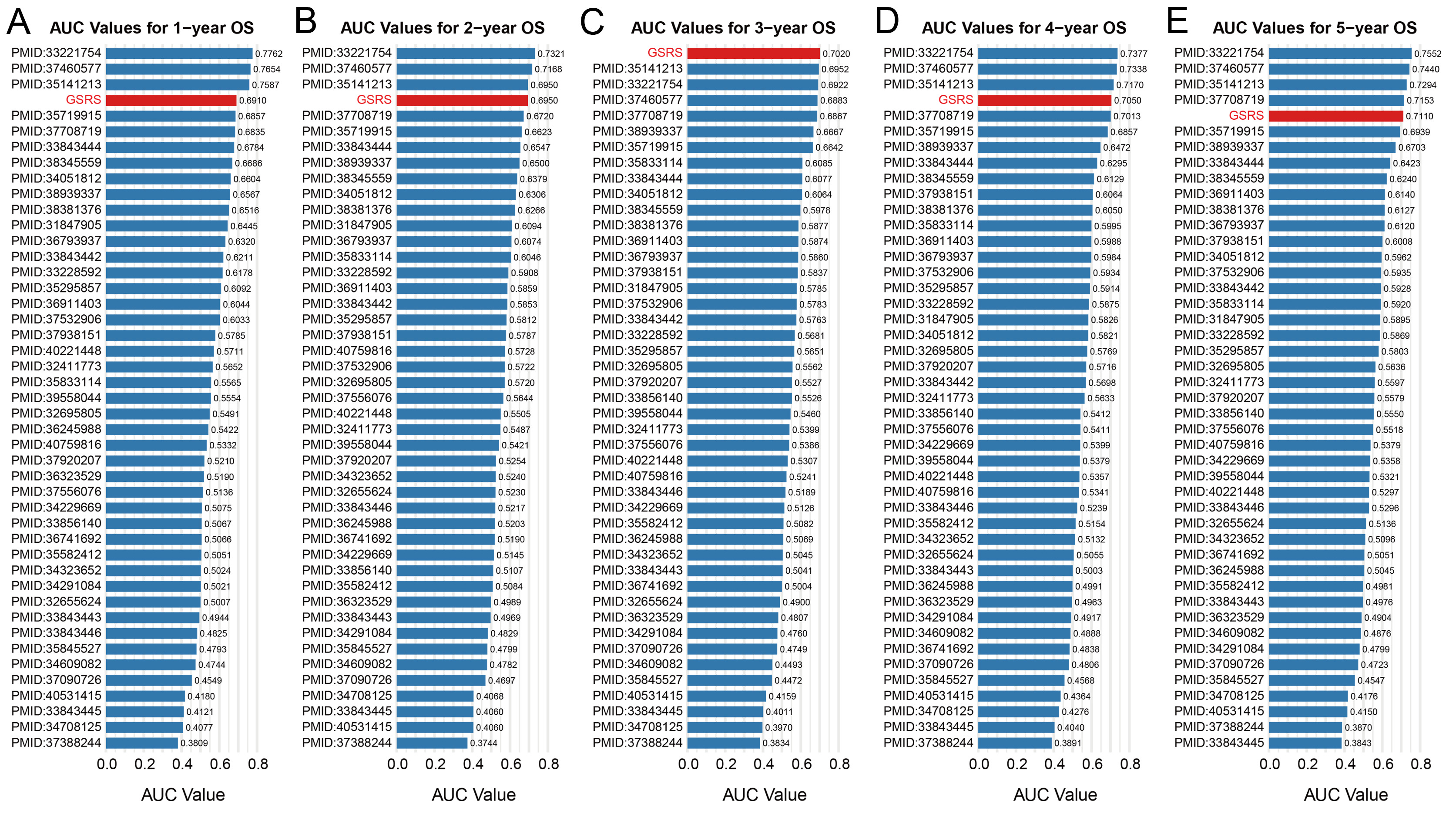

Supplement: S22 Fig — (TIF) [file pone.0334104.s022.tif]
