# Supplementary material for: Developing and experimentally validating a glucocorticoid signaling-related gene signature to evaluate the prognosis and immunotherapeutic response in kidney renal clear cell carcinoma
Source: PLoS One. 2025 Oct 13;20(10):e0334104. doi: 10.1371/journal.pone.0334104 (PMC12517536; doi:10.1371/journal.pone.0334104)
Supplement: S1 File — (DOCX) [file pone.0334104.s044.docx]

**Detailed materials and methods**

Content

[1. Bioinformatical analyses section 1](#_Toc187431410)

[1.1 Data collection and processing 1](#_Toc187431411)

[1.2 Unsupervised clustering 2](#_Toc187431412)

[1.3 Genomic difference detection 2](#_Toc187431413)

[1.4 Protein-protein interaction (PPI) network analysis 2](#_Toc187431414)

[1.5 Functional enrichment 3](#_Toc187431415)

[1.6 Feature selection and risk signature construction 3](#_Toc187431416)

[1.7 Detecting the association of identified genes with malignant phenotypes of KIRC at a single-cell level 3](#_Toc187431417)

[1.8 Single-sample gene set enrichment analysis (ssGSEA) 3](#_Toc187431418)

[1.9 Evaluating immune cells infiltration proportion in KIRC samples 4](#_Toc187431419)

[1.10 Spatial transcriptomics analyses 4](#_Toc187431420)

[1.11 Meta-analyses 4](#_Toc187431421)

[1.12 Evaluating immunotherapeutic effectiveness 4](#_Toc187431422)

[1.13 Drug sensitivity analyses 5](#_Toc187431423)

[2. Experimental studies section 5](#_Toc187431424)

[2.1 Sample collection 5](#_Toc187431425)

[2.2 Enzyme linked immunosorbent assay (ELISA) 5](#_Toc187431426)

[2.3 Immunofluorescence analyses 6](#_Toc187431427)

[2.4 Cell culture 6](#_Toc187431428)

[2.5 Lentiviral-mediated Nfkb2 knockdown in Renca cells 7](#_Toc187431429)

[2.6 RT-qPCR 7](#_Toc187431430)

[2.7 Establishment of subcutaneous tumor model in BALB/c mice 7](#_Toc187431431)

[2.8 Immunohistochemistry (IHC) 8](#_Toc187431432)

[3. Statistical analyses 8](#_Toc187431433)

[4. References 9](#_Toc187431434)

# Bioinformatical analyses section

## 1.1 Data collection and processing

The transcriptome sequencing data of 72 paracarcinoma and 535 kidney renal clear cell carcinoma (KIRC) samples, along with their corresponding clinicopathological and overall survival (OS) follow-up information, were obtained from The Cancer Genome Atlas (TCGA, https://portal.gdc.cancer.gov/) to serve as the training dataset. The RNA sequencing (RNA-seq) data from the TCGA-KIRC cohort were downloaded in both count and Fragments Per Kilobase of exon model per Million mapped fragments (FPKM) formats. For validation, the GSE29609 dataset (1), which includes transcriptome microarray sequencing data from 39 KIRC samples along with their clinicopathological features and OS information, was retrieved from the Gene Expression Omnibus (GEO, https://ncbi.nlm.nih.gov/geo/). Additionally, the GSE167573 dataset (2), containing RNA-seq data from 14 paracarcinoma and 63 KIRC tissues along with corresponding clinicopathological features and OS information, was also obtained from GEO. Furthermore, we queried the ArrayExpress database (https://www.ebi.ac.uk/biostudies/arrayexpress) and included the E-MTAB-1980 dataset (3), which comprises transcriptome microarray sequencing data from 101 KIRC samples, in our validation datasets. The clinicopathological and OS information for the E-MTAB-1980 cohort were sourced from the supplementary materials of its original publication (3). The GSE29609, GSE167573, and E-MTAB-1980 datasets were utilized to validate the predictive capability of the risk signature for OS in KIRC patients. All RNA-seq data were converted to Transcripts Per Kilobase of exon model per Million mapped reads (TPM) format and subsequently transformed using "log2(TPM +1)". The sva package was employed to minimize batch effects across these datasets. Cases with follow-up durations of less than 30 days and genes with an average expression value below 0.5 were excluded from the analysis. The baseline clinicopathological features of the TCGA-KIRC, GSE29609, GSE167573, and E-MTAB-1980 cohorts can be found in **Table S1**.

To further investigate the immunogenetic associations of the risk signature, additional KIRC public cohorts were incorporated. The GSE53757 dataset (4), which includes transcriptome microarray sequencing data from 72 paracarcinoma and 72 KIRC samples, the GSE73731 dataset (5), containing transcriptome microarray sequencing data from 265 KIRC samples, and the GSE40435 dataset (6), which includes transcriptome microarray sequencing data from 101 paracarcinoma and 101 KIRC samples, were downloaded from GEO for verification purposes.

The JAVELIN Renal 101 trial, a phase III clinical study, was designed to assess the efficacy and safety of avelumab (an anti-PD-L1 agent) in combination with axitinib (a tyrosine kinase inhibitor) for the treatment of advanced KIRC. The control group in this trial received sunitinib (another tyrosine kinase inhibitor), with the primary endpoint being progression-free interval (PFI). RNA-seq data from 726 KIRC samples, along with their corresponding PFI and response statuses to avelumab plus axitinib, were obtained from the study conducted by R. J. Motzer et al (7).

In addition to KIRC, this study also encompassed pan-cancer analyses, including 32 other cancer types. RNA-seq data and OS information for these cancers were downloaded from TCGA. Like the KIRC data, all RNA-seq data were transformed into "log2(TPM+1)" format, and cases with follow-up durations of less than 30 days were excluded from the analysis.

A comprehensive set of 655 glucocorticoid signaling-related genes was systematically curated from the Molecular Signatures Database (MSigDB, <https://www.gsea-msigdb.org/gsea/msigdb/index.jsp>), with annotation details provided in **Table S2**. These genes encompass multiple functional categories, including glucocorticoid biosynthesis, metabolic regulation, secretory pathways, receptor-mediated signaling transduction, and human physiological responses to glucocorticoid treatment.

## 1.2 Unsupervised clustering

Non-negative matrix factorization (NMF) clustering was performed to investigate the potential association between the expression profiles of glucocorticoid signaling-related genes and OS in TCGA-KIRC patients. The analysis was implemented using the NMF R package, employing Kullback-Leibler divergence as the objective function for dimensionality reduction. To ensure robust clustering, the optimal number of clusters was determined through a comprehensive evaluation of three distinct metrics: cophenetic correlation coefficients, dispersion coefficients, and silhouette scores, as previously reported (8).

## 1.3 Genomic difference detection

Differential gene expression analysis between the two groups was performed using the edgeR package in R software. Statistically significant differentially expressed genes (DEGs) were identified based on stringent filtering criteria: an absolute log2 fold change (|log2FC|) threshold of >1 and a false discovery rate (FDR) adjusted P-value of <0.05, following Benjamini-Hochberg correction for multiple testing.

## 1.4 Protein-protein interaction (PPI) network analysis

To elucidate potential functional interactions among the identified genes, a PPI network was constructed using the STRING database (<https://string-db.org/>) (9). The network was generated with a medium confidence interaction score threshold of 0.4, which represents the minimum required combined score for including protein interactions in the analysis (10, 11).

## 1.5 Functional enrichment

Functional enrichment analysis of the identified genes was performed using the Metascape platform (<https://metascape.org/>) (12). Gene Ontology (GO) biological processes, Kyoto Encyclopedia of Genes and Genomes (KEGG) pathways, and Reactome pathways were systematically analyzed. Enrichment terms with a nominal P-value < 0.05 and a Benjamini-Hochberg adjusted P-value (q-value) < 0.05 were considered statistically significant. The analysis incorporated multiple testing correction and employed default parameters, including a minimum overlap of 3 genes and an enrichment factor > 1.5.

## 1.6 Feature selection and risk signature construction

To identify genes significantly associated with OS in the TCGA-KIRC cohort, we employed a dual-feature selection approach using both least absolute shrinkage and selection operator (LASSO) regression and random forest algorithms. LASSO regression analysis with 10-fold cross-validation was performed using the glmnet package in R, while random forest analysis was conducted using the RandomForestSRC R package. Genes consistently identified by both LASSO regression and random forest analysis were subsequently incorporated into the risk signature.

Using the mRNA expression levels of these identified genes, we constructed a multivariate Cox regression model to assess OS in TCGA-KIRC patients, implemented through the survival R package. Based on this multivariate Cox regression model, we calculated a glucocorticoid signaling-related score (GSRS) for each KIRC patient using the following formula: $GSRS=\sum_{i=1}^{n} ({Coefficient}_{i}*{exp(gene)}_{i}$, where "Coefficient" represents the regression coefficient of each gene in the multivariate Cox regression model, and "exp(gene)" denotes the corresponding gene mRNA expression level.

## 1.7 Detecting the association of identified genes with malignant phenotypes of KIRC at a single-cell level

To elucidate the functional relevance of the identified genes in renal cell carcinoma progression, we performed single-cell level analyses using the CancerSEA database (<http://biocc.hrbmu.edu.cn/CancerSEA/>) (13). This comprehensive platform enabled us to systematically investigate the correlation between gene expression profiles and critical malignant phenotypes, including epithelial-mesenchymal transition (EMT), angiogenesis, proliferation, metastasis, and stemness characteristics, specifically in KIRC samples.

## 1.8 Single-sample gene set enrichment analysis (ssGSEA)

To assess the activation status of glucocorticoid signaling and immune-related pathways, ssGSEA was performed using the GSVA package in R software. The analysis was conducted with default parameters, and the enrichment scores were normalized across all samples to ensure comparability.

## 1.9 Evaluating immune cells infiltration proportion in KIRC samples

The infiltration levels of 22 distinct immune cell types in KIRC samples were quantitatively assessed using the CIBERSORT-ABS algorithm. To further validate the correlation between GSRS and CD8+ T cell infiltration, we employed five additional deconvolution algorithms: Tumor Immune Estimation Resource (TIMER), CIBERSORT, QUANTISEQ, MCP-COUNTER, and xCELL, to systematically evaluate the infiltration proportions of CD8+ T cells, which were conducted using the immunedeconv R package (14).

## 1.10 Spatial transcriptomics analyses

Spatial transcriptomics analysis was performed using the CROST platform (https://ngdc.cncb.ac.cn/crost/home) (15) to systematically investigate the spatial correlation between the expression patterns of identified genes and CD8+ T cell infiltration. The spatial transcriptomics data were acquired from the published dataset by Meylan M et al. (16), which provided comprehensive spatial gene expression profiles across KIRC tissues. Cell type annotations and expressions of ACADM, ANGPTL4, NFKB2, and CD8A in KIRC samples were directly obtained from the CROST platform. Cell type annotations for the spatial transcriptomics data were obtained from matched scRNA-seq data. To analyze the spatial correlation between GSRS and TEX, we additionally downloaded spatial transcriptomic data from KIRC samples published by Meylan M et al. via the same platform. Data preprocessing was conducted using the Seurat package in R, including filtering out low-quality spots (fewer than 200 genes detected or mitochondrial gene content >20%). The gene expression matrix was normalized using SCTransform to mitigate technical variation. GSRS scores were computed for each spot using the established formula, while TEX status was evaluated based on combined CD8A and PDCD1 expression levels. All results were visualized with ggplot2.

## 1.11 Meta-analyses

Meta-analyses were performed using the meta package in R software to synthesize effect estimates, specifically hazard ratios (HRs), odds ratios (ORs), and correlation coefficients. Heterogeneity across studies was assessed using the Cochrane Q test, with statistical significance set at P < 0.05. When significant heterogeneity was detected (P < 0.05), a random-effects model was employed. In the absence of significant heterogeneity (P ≥ 0.05), a fixed-effect model was utilized to provide more precise effect estimates.

## 1.12 Evaluating immunotherapeutic effectiveness

The therapeutic response of KIRC patients to immune checkpoint inhibitors (ICIs), including anti-PD1 and anti-PDL1 agents, was systematically assessed using the Tumor Immune Dysfunction and Exclusion (TIDE) algorithm (http://tide.dfci.harvard.edu/). The TIDE scoring system demonstrated that elevated scores were significantly correlated with an increased probability of clinical benefit from ICI-based immunotherapy regimens. The transcriptome sequencing data of the KIRC samples was normalized by R software.

## 1.13 Drug sensitivity analyses

We employed the oncoPredict R package, a robust computational tool for drug sensitivity prediction, to systematically evaluate the half-maximal inhibitory concentration (IC50) values of KIRC subjects in response to various chemotherapeutic agents. This analysis was performed based on the Genomics of Drug Sensitivity in Cancer (GDSC) database, which provides comprehensive drug response data across multiple cancer cell lines (17).

# 2. Experimental studies section

## 2.1 Sample collection

The paracarcinoma tissues, KIRC samples, and peripheral blood samples from KIRC patients were collected from the Shenzhen Third People's Hospital between March 2024 and December 2024. Considering the influence of circadian rhythms on serum glucocorticoid levels, blood draws were performed in the morning for all participants. The inclusion criteria for KIRC patients were as follows: 1) Postoperative pathological diagnosis of KIRC; 2) No preoperative chemotherapy, radiotherapy, or immunotherapy; 3) No long-term use of glucocorticoids prior to surgery; 4) Signed informed consent. The exclusion criteria were: 1) Severe endocrine disorders; 2) Metabolic diseases; 3) Heart failure or other severe cardiovascular diseases; 4) Autoimmune diseases; 5) Long-term use of medications such as ketoconazole, erythromycin, clarithromycin, estrogen, oral contraceptives, phenytoin, phenobarbital, glutethimide, carbamazepine, primidone, rifampin, ephedrine, antacids, cholestyramine, or gemfibrozil affecting serum glucocorticoid concentrations. Ultimately, 21 KIRC patients were enrolled, and their baseline clinicopathological characteristics are detailed in **Table S1**. The optimal age stratification threshold was determined using X-tile software analysis (18) of the TCGA-KIRC cohort.

To minimize selection bias and potential confounding effects, we established a comparison cohort at a 1:2 ratio to assess differences in serum glucocorticoid levels between KIRC patients and healthy donors. Peripheral blood samples from healthy donors were obtained from the Physical Examination Center of the Shenzhen Third People's Hospital. The inclusion criteria for healthy donors were: 1) No cancer diagnosis; 2) Signed informed consent; 3) No long-term use of glucocorticoids. The exclusion criteria were: 1) Metabolic diseases such as diabetes and gout; 2) Heart failure or other severe cardiovascular diseases; 3) Severe endocrine disorders such as Cushing's syndrome; 4) Autoimmune diseases; 5) Severe kidney disease; 6) Long-term use of medications such as ketoconazole, erythromycin, clarithromycin, estrogen, oral contraceptives, phenytoin, phenobarbital, glutethimide, carbamazepine, primidone, rifampin, ephedrine, antacids, cholestyramine, or gemfibrozil affecting serum glucocorticoid concentrations. Finally, 42 healthy donors were included in this study. The protocol of this study has been reviewed and approved by the Ethics Committee of Shenzhen Third People's Hospital.

## 2.2 Enzyme linked immunosorbent assay (ELISA)

Peripheral blood samples were collected from human participants using standard blood collection tubes without anticoagulants. For murine subjects, blood was drawn from the orbital sinus and subsequently transferred into anticoagulant-free collection tubes (HYHC0640; Huayunbio, China). All blood samples were allowed to clot naturally at ambient temperature (approximately 25°C) for 30 minutes before being centrifuged at 2000 × g for 10 minutes to separate the serum fraction. The resulting serum supernatant was promptly aliquoted and stored at -80°C until analysis.

Serum glucocorticoid levels were quantified using species-specific ELISA kits according to the manufacturers' protocols. Human serum samples were analyzed for cortisol concentration using a Human Cortisol ELISA Kit (E-OSEL-H0006; Elabscience, China), while mouse serum samples were assessed for corticosterone (CORT) levels using a Corticosterone ELISA Kit (ab108821; Abcam, UK). These analytes represent the predominant circulating glucocorticoids in their respective species. The ELISA procedures, including plate preparation, standard curve generation, sample incubation, and optical density measurement, were performed as previously described in our published methodology (19), with all samples and standards run in triplicate to ensure analytical precision.

## 2.3 Immunofluorescence analyses

Freshly excised KIRC and paracarcinoma tissues from both BALB/c mice and KIRC patients were immediately fixed in 4% paraformaldehyde (PFA) for 24 hours at 4°C. Following fixation, tissues were dehydrated through a graded series of ethanol, cleared in xylene, and embedded in paraffin. Sections of 5 µm thickness were cut using a microtome and mounted onto poly-L-lysine-coated glass slides.

Paraffin-embedded tissue sections were deparaffinized in xylene and rehydrated through a descending ethanol series. Antigen retrieval was performed by heating the sections in 10 mM sodium citrate buffer (pH 6.0) at 95°C for 20 minutes, followed by cooling to room temperature. Sections were permeabilized with 0.1% Triton X-100 in phosphate-buffered saline (PBS) for 10 minutes and blocked with 5% bovine serum albumin (BSA) in PBS for 1 hour at room temperature to reduce non-specific binding.

For the human tissues, primary antibodies (anti-NFKB2, 1:200, A19605; anti-ANGPTL4, 1:200, A2011; anti-ACADM, 1:200, A4567; ABclonal, China) were applied and incubated overnight at 4°C. After washing with PBS, sections were incubated with species-specific secondary antibodies conjugated to Alexa Fluor® 488 (1:500, Abcam, UK) for 1 hour at room temperature.

For the tumor tissues extracted from BALB/c mice, the slides were then incubated overnight at 4°C with primary antibodies: mouse anti-CD8 (1:200, ab217344; Abcam, UK) and rabbit anti-PDCD1 (1:200, A19135; ABclonal, China). After washing three times with PBS, the sections were incubated with secondary antibodies: Alexa Fluor® 488-conjugated goat anti-rabbit IgG and Alexa Fluor® 594-conjugated goat anti-mouse IgG for 1 hour at room temperature in the dark.

Nuclei were counterstained with 4',6-diamidino-2-phenylindole (DAPI,
RM02978; ABclonal, China) (1 µg/mL) for 5 minutes. Fluorescence images were captured using a confocal laser scanning microscope (Zesis, Germany) and at least five randomly selected fields per section were selected for quantitative analyses.

## 2.4 Cell culture

The Renca cell line, a murine KIRC cell line, was purchased from the American Tissue Culture Collection (ATCC). Cells were maintained in Dulbecco's Modified Eagle Medium (DMEM) supplemented with 10% fetal bovine serum (FBS), 2 mM L-glutamine, 100 U/mL penicillin, and 100 µg/mL streptomycin. The cells were incubated at 37°C in a humidified atmosphere containing 5% CO_2_.

## 2.5 Lentiviral-mediated Nfkb2 knockdown in Renca cells

The lentiviral vector encoding shRNA targeting Nfkb2 (sh-Nfkb2) and a control vector encoding scrambled shRNA (sh-Ctrl) were synthesized by HANBIO (Shanghai, China). The sequences of sh-Nfkb2 and sh-Ctrl are shown as follows: sh-Nfkb2: Sense: 5'-CCGGCCTGTCTAATCGAAATCTTATTTCAAGAGAATAAGATTTCGATTAGACAGGTTTTTG-3', Antisense: 3'-GGGACAGATTAGCTTTAGAATAAAGTTCTCTTATTCTAAAGCTAATCTGTCCAAAAACCTTT-5'; sh-Ctrl: Sense: 5'-CCGGGATTAACTTACTGTCTATCAATTCAAGAGATTGATAGACAGTAAGTTAATC-TTTTTG-3', Antisense: 3'-GCTAATTGAATGACAGATAGTTAAGTTCTCTAACTATCTGTCATTCAATTAGAAAAACCTTT-5'. The vectors also contained a puromycin resistance gene for selection. Renca cells were seeded in 6-well plates at a density of 1 × 10^5^ cells/well in complete DMEM medium and incubated overnight at 37°C with 5% CO_2_. After 24 hours, cells were transduced with lentiviral particles (sh-Nfkb2 or sh-Ctrl) at a multiplicity of infection (MOI) of 10 in the presence of 8 μg/mL Polybrene. The medium was replaced with fresh complete medium 24 hours post-transduction. Transduced cells were selected with 2 μg/mL puromycin for 7 days. The surviving cells were considered stable Nfkb2 knockdown or control Renca cells. Total RNA was extracted from the selected cells. Nfkb2 mRNA levels were assessed by real-time quantitative PCR (RT-qPCR) to confirm knockdown efficiency.

## 2.6 RT-qPCR

Total RNA was extracted from Renca cells using TRIzol reagent (Invitrogen, USA) according to the manufacturer’s instructions. RNA concentration and purity were determined using a NanoDrop spectrophotometer (Thermo Fisher Scientific, USA). First-strand cDNA was synthesized from 1 µg of total RNA using a PrimeScript RT Reagent Kit (RR047A; Takara, Japan) with oligo(dT) primers, following the manufacturer’s protocol. qPCR was performed using SYBR Green Master Mix (Roche, Switzerland) on a LightCycler 480 II system (Roche, Switzerland). Gapdh was used as an internal control and the primer sequences of Nfkb2 and Gapdh can be found in **Table S3**. Relative mRNA expression levels of NF-κB2 were calculated using the 2^(-ΔΔCt) method, normalized to Gapdh. Data are presented as mean ± SD from three independent experiments.

## 2.7 Establishment of subcutaneous tumor model in BALB/c mice

12 male BALB/c mice, aged 6-7 weeks, were procured from the Guangdong Medical Laboratory Animal Center. To establish a subcutaneous tumor model, 1 × 10^6^ Renca cells, which had been transfected with either an empty vector plasmid (vector [KD]) or an NFKB2 shRNA plasmid (KD-NFKB2), were inoculated into the right flank of each mouse. In the corticosterone (CORT) treatment group, mice bearing NFKB2 knockdown Renca cells were administered drinking water supplemented with 50 μg/mL CORT (C-117; Sigma-Aldrich, USA) (20). Tumor progression was assessed every three days using a digital caliper, and tumor volume was determined using the formula: V = 0.52 × L × W^2^, where "L" denotes the tumor's longest diameter and "W" represents the longest transverse diameter perpendicular to "L". The experiment was concluded when the tumor's longest diameter reached approximately 1.5 cm, at which point the mice were humanely euthanized by cervical dislocation. No mortality occurred prior to the experimental endpoint. This study received approval from the Experimental Animal Ethics Committee of Southern Medical University.

## 2.8 Immunohistochemistry (IHC)

The steps of embedding, slicing, antigen repair, etc. are the same as those in the immunofluorescence analysis section mentioned above. For IHC analyses, the sections were then incubated overnight at 4°C with primary antibodies against Ki67 (1:200, A20018; ABclonal, China) and Nfkb2 (1:200, A19605; ABclonal, China). After washing with PBS, the sections were incubated with a biotinylated secondary antibody (AS014; ABclonal, China) for 1 hour at room temperature. The immunoreactivity was visualized using the avidin-biotin-peroxidase complex (ABC) method with 3,3'-diaminobenzidine (DAB) as the chromogen (Vector Laboratories). The sections were counterstained with hematoxylin, dehydrated, and mounted with a coverslip.The immunohistochemical staining intensity was quantitatively analyzed using Image-Pro Plus software (Media Cybernetics, USA). The mean integrated optical density (IOD) was measured for each stained section to assess the expression levels of Ki67 and Nfkb2. Five randomly selected fields per section were analyzed at 200× magnification, and the mean IOD values were calculated for statistical analysis.

# Statistical analyses

All statistical analyses were conducted using R software (version 4.3.0, Austria). Continuous variables were summarized as mean ± standard deviation (SD), while categorical variables were expressed as frequencies and percentages (n [%]). For comparisons of continuous variables between groups, the Welch’s corrected t-test or Wilcoxon signed-rank test was employed, depending on the data distribution and variance assumptions. Categorical variables were compared using the Chi-square test or Fisher’s exact test, as appropriate. Univariate and multivariate Cox regression analyses were performed using the survival package to assess the association between variables and outcomes. Differences in OS over the follow-up period were evaluated using Kaplan-Meier curves, with statistical significance assessed via the log-rank test or two-stage hazard rate comparison. Dimensionality reduction techniques, including principal component analysis (PCA) and t-distributed stochastic neighbor embedding (t-SNE), were implemented using the prcomp function and the Rtsne package, respectively. Spearman correlation coefficients were calculated using the cor.test function to evaluate the strength and direction of associations between variables. The time-dependent receiver operating characteristic (ROC) analyses were conducted using the timeROC R package. Unless otherwise stated, all statistical tests were two-sided, and a p-value < 0.05 was considered statistically significant.

# References

1. Edeline J, Mottier S, Vigneau C, Jouan F, Perrin C, Zerrouki S, et al. Description of 2 angiogenic phenotypes in clear cell renal cell carcinoma. Hum Pathol. 2012;43(11):1982-90.

2. Sun G, Chen J, Liang J, Yin X, Zhang M, Yao J, et al. Integrated exome and RNA sequencing of TFE3-translocation renal cell carcinoma. Nat Commun. 2021;12(1):5262.

3. Sato Y, Yoshizato T, Shiraishi Y, Maekawa S, Okuno Y, Kamura T, et al. Integrated molecular analysis of clear-cell renal cell carcinoma. Nat Genet. 2013;45(8):860-7.

4. von Roemeling CA, Radisky DC, Marlow LA, Cooper SJ, Grebe SK, Anastasiadis PZ, et al. Neuronal pentraxin 2 supports clear cell renal cell carcinoma by activating the AMPA-selective glutamate receptor-4. Cancer Res. 2014;74(17):4796-810.

5. Wei X, Choudhury Y, Lim WK, Anema J, Kahnoski RJ, Lane B, et al. Recognizing the Continuous Nature of Expression Heterogeneity and Clinical Outcomes in Clear Cell Renal Cell Carcinoma. Sci Rep. 2017;7(1):7342.

6. Wozniak MB, Le Calvez-Kelm F, Abedi-Ardekani B, Byrnes G, Durand G, Carreira C, et al. Integrative genome-wide gene expression profiling of clear cell renal cell carcinoma in Czech Republic and in the United States. PLoS One. 2013;8(3):e57886.

7. Motzer RJ, Robbins PB, Powles T, Albiges L, Haanen JB, Larkin J, et al. Avelumab plus axitinib versus sunitinib in advanced renal cell carcinoma: biomarker analysis of the phase 3 JAVELIN Renal 101 trial. Nat Med. 2020;26(11):1733-41.

8. Huang X, Bajpai AK, Sun J, Xu F, Lu L, Yousefi S. A new gene-scoring method for uncovering novel glaucoma-related genes using non-negative matrix factorization based on RNA-seq data. Front Genet. 2023;14:1204909.

9. Szklarczyk D, Kirsch R, Koutrouli M, Nastou K, Mehryary F, Hachilif R, et al. The STRING database in 2023: protein-protein association networks and functional enrichment analyses for any sequenced genome of interest. Nucleic Acids Res. 2023;51(D1):D638-D46.

10. Li C, Zhang L, Zhang L, Zhang G. Correlation between elevated HCLS1 levels and heart failure: A diagnostic biomarker. Medicine (Baltimore). 2024;103(23):e38484.

11. Zhang Y, Niu Y, Peng Y, Pan X, Wang F. COL3A1, COL5A1 and COL6A2 serve as potential molecular biomarkers for osteoarthritis based on weighted gene co‑expression network analysis bioinformatics analysis. Exp Ther Med. 2023;26(5):540.

12. Zhou Y, Zhou B, Pache L, Chang M, Khodabakhshi AH, Tanaseichuk O, et al. Metascape provides a biologist-oriented resource for the analysis of systems-level datasets. Nat Commun. 2019;10(1):1523.

13. Yuan H, Yan M, Zhang G, Liu W, Deng C, Liao G, et al. CancerSEA: a cancer single-cell state atlas. Nucleic Acids Res. 2019;47(D1):D900-D8.

14. Sturm G, Finotello F, List M. Immunedeconv: An R Package for Unified Access to Computational Methods for Estimating Immune Cell Fractions from Bulk RNA-Sequencing Data. Methods Mol Biol. 2020;2120:223-32.

15. Wang G, Wu S, Xiong Z, Qu H, Fang X, Bao Y. CROST: a comprehensive repository of spatial transcriptomics. Nucleic Acids Res. 2024;52(D1):D882-D90.

16. Meylan M, Petitprez F, Becht E, Bougouin A, Pupier G, Calvez A, et al. Tertiary lymphoid structures generate and propagate anti-tumor antibody-producing plasma cells in renal cell cancer. Immunity. 2022;55(3):527-41 e5.

17. Maeser D, Gruener RF, Huang RS. oncoPredict: an R package for predicting in vivo or cancer patient drug response and biomarkers from cell line screening data. Brief Bioinform. 2021;22(6).

18. Camp RL, Dolled-Filhart M, Rimm DL. X-tile: a new bio-informatics tool for biomarker assessment and outcome-based cut-point optimization. Clin Cancer Res. 2004;10(21):7252-9.

19. Shi X, Pang S, Zhou J, Yan G, Gao R, Wu H, et al. Bladder-cancer-derived exosomal circRNA_0013936 promotes suppressive immunity by up-regulating fatty acid transporter protein 2 and down-regulating receptor-interacting protein kinase 3 in PMN-MDSCs. Mol Cancer. 2024;23(1):52.

20. Wang Y, Su J, Zhou P, Pan XY, Huang GX, Yin LJ, et al. Glucocorticoids promote lung metastasis of pancreatic cancer cells through enhancing cell adhesion, migration and invasion. Endocr J. 2023;70(7):731-43.
